# From Conventional to Precision Therapy in Canine Mammary Cancer: A Comprehensive Review

**DOI:** 10.3389/fvets.2021.623800

**Published:** 2021-02-17

**Authors:** Guillermo Valdivia, Ángela Alonso-Diez, Dolores Pérez-Alenza, Laura Peña

**Affiliations:** ^1^Department Animal Medicine, Surgery and Pathology, Veterinary School, Complutense University of Madrid, Madrid, Spain; ^2^Mammary Oncology Unit, Complutense Veterinary Teaching Hospital, Complutense University of Madrid, Madrid, Spain

**Keywords:** canine mammary cancer, targeted therapy, markers, immunotherapy, immunophenotyping, hormonal therapy, tyrosine kinase receptors inhibitors

## Abstract

Canine mammary tumors (CMTs) are the most common neoplasm in intact female dogs. Canine mammary cancer (CMC) represents 50% of CMTs, and besides surgery, which is the elective treatment, additional targeted and non-targeted therapies could offer benefits in terms of survival to these patients. Also, CMC is considered a good spontaneous intermediate animal model for the research of human breast cancer (HBC), and therefore, the study of new treatments for CMC is a promising field in comparative oncology. Dogs with CMC have a comparable disease, an intact immune system, and a much shorter life span, which allows the achievement of results in a relatively short time. Besides conventional chemotherapy, innovative therapies have a large niche of opportunities. In this article, a comprehensive review of the current research in adjuvant therapies for CMC is conducted to gather available information and evaluate the perspectives. Firstly, updates are provided on the clinical–pathological approach and the use of conventional therapies, to delve later into precision therapies against therapeutic targets such as hormone receptors, tyrosine kinase receptors, *p*53 tumor suppressor gene, cyclooxygenases, the signaling pathways involved in epithelial–mesenchymal transition, and immunotherapy in different approaches. A comparison of the different investigations on targeted therapies in HBC is also carried out. In the last years, the increasing number of basic research studies of new promising therapeutic agents on CMC cell lines and CMC mouse xenografts is outstanding. As the main conclusion of this review, the lack of effort to bring the *in vitro* studies into the field of applied clinical research emerges. There is a great need for well-planned large prospective randomized clinical trials in dogs with CMC to obtain valid results for both species, humans and dogs, on the use of new therapies. Following the One Health concept, human and veterinary oncology will have to join forces to take advantage of both the economic and technological resources that are invested in HBC research, together with the innumerable advantages of dogs with CMC as a spontaneous animal model.

## Introduction

Canine mammary tumors (CMTs) are a highly heterogeneous group of neoplasms that represent between 50 and 70% of all tumors in intact female dogs (1–4). The prevalence varies depending on the geographic location, being greater in countries where ovariectomy is not routinely performed (4). In these countries, the prevalence of mammary neoplasms in female dogs is three times higher than the prevalence in women (3). Historically, roughly 50% of CMTs are considered to be malignant (1, 2, 5, 6). However, recent studies have shown an increase in malignant *vs*. benign tumors over the last years, a similar trend that has been detected in human medicine (2). Canine mammary cancer (CMC) and human breast cancer (HBC) share not only the aforesaid trend but also many epidemiological, environmental, biological, clinical, genetic, and pathological features, including a remarkable histological and molecular heterogeneity. Many authors have claimed CMC as a good spontaneous model for the study of HBC, especially the inflammatory mammary cancer, the deadliest type (7–12). In female dogs and in women, mammary cancer is the most frequently diagnosed malignancy (4) and the leading cause of cancer-related death in women worldwide (13). For this reason, over the past two decades, innovative HBC treatments have incredibly evolved to place emphasis on more molecularly directed individual therapies while diminishing radio- and chemotherapy to reduce the adverse effects of treatment (14). Since the publication of the intrinsic molecular classification of Perou and Solie in 2000, which distinguished four subtypes of HBC (luminal A, luminal B, basal-like, and HER-2 enriched), the clinical management shifted to a biology-centered approach, based on the expressions of estrogen and progesterone receptors (luminal subtypes), the human epidermal growth factor receptor 2 (HER-2), and basal markers (positive for high-molecular-weight cytokeratins, as cytokeratins 5/6, 14, and 17) (15). Nowadays, the classification of five molecular subtypes (luminal A, luminal B HER-2–, luminal B HER-2+, HER-2 enriched, and triple negative) is the most widely utilized in human medicine to elect targeted therapy: anti-estrogenic drugs for the luminal subtypes and anti-HER-2 treatment for the HER-2-enriched tumors. Unfortunately, since triple-negative mammary cancer does not currently have a specific targeted therapy, its prognosis is poor (14). Surgery is the treatment of choice in both HBC and CMC, and adjuvant therapies are only given on a routine basis in HBC. On the contrary to HBC treatment, adjuvant chemotherapy has not been proven to have a clear benefit in dogs with CMC yet (16–19). In spite that the application of clinical staging of patients with CMC and the histological grading of the neoplasms have helped to standardize prognosis and treatments, no precision therapies are routinely administered to dogs bearing mammary neoplasms. For all these reasons, the CMC-related mortality is relatively high: over 40% of the patients die within a year after diagnosis (20). There is an urge to developing updated therapeutic protocols and targeted therapies. Therefore, a comprehensive review of the current research in adjuvant therapies for CMC is conducted here to gather available information and evaluate the perspectives.

## Conventional and New Clinical and Histological Approaches

### Staging System

Dogs with CMC are staged according to the modified World Health Organization (WHO) TNM system (T, size of tumor; N, affectation of lymph nodes; M, distant metastasis) (21).

More recently, a pathological staging system inspired in human oncology, in which T is replaced by pathologic tumor size (pT) and N is replaced by the pathologic nodal status (pN), with the addition of lymphovascular invasion (LVI) has been proposed, the stages being 0, I, II, IIIA, and IIIB (22). The use of this system is still limited, the major criticism being its inability to discriminate different stages of malignancy in cases with no LVI or lymph node affectation, which are the vast majority.

The current staging system is not flexible enough to allow prognostic differences between specific tumor types and subtypes and does not consider tumor grades or lymphovascular invasion (21). Therefore, new “bio-score” systems combining the anatomical staging (TNM) with many histological and biological variables have been developed: multivariate scoring (scores from 0 to 40) and refined flexible scoring (scores from 0 to 6.5). Although both systems were accurate in predicting the survival, the refined flexible scoring was superior in differentiating dogs with a high or a low risk of metastasis (23). Greater efforts should be made to implement these bio-score systems in larger prospective studies, allowing for more widespread use and the refinement of these increasingly accurate prognostic systems.

### Histopathological Classification and Grading

The new classification of CMTs (24), which substitutes the WHO's 1974 and the last 2011 classification (5), together with the current grading system used worldwide for malignant CMC (9), as an adaptation of the Nottingham method utilized for HBC (25), have given tools to pathologists for accurate diagnosis and prognosis of CMTs since several studies have validated the prognostic significance of histopathological classification and grading (26, 27).

### Cell Markers for Diagnosis: Immunophenotyping

**Figure 1:**
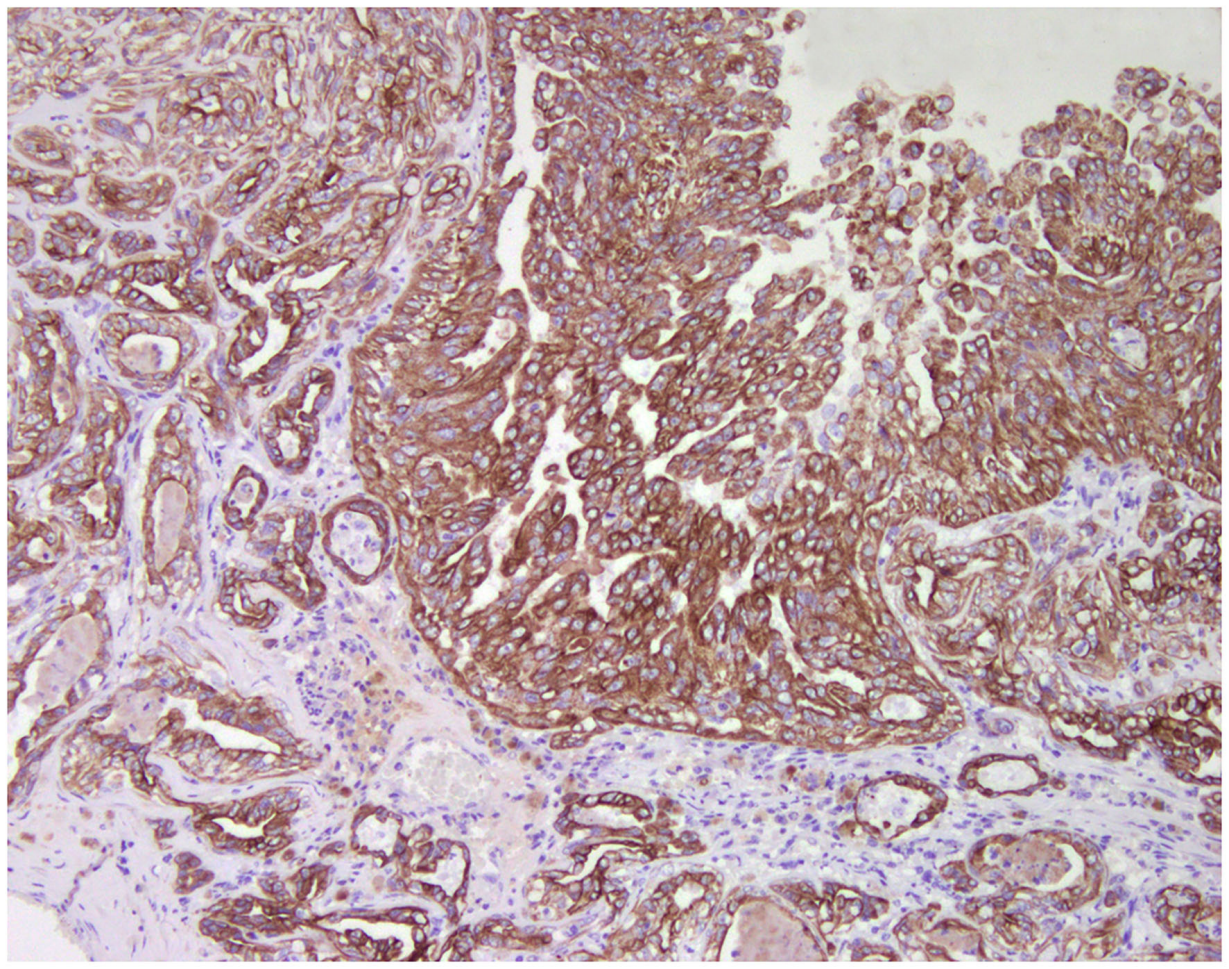
Tubulo-papillary carcinoma, mammary gland, dog. Immunohistochemical cytoplasmic staining of wide-spectrum cytokeratins (*AE1*/*AE3*). Luminal and basal/myoepithelial cells are positive (*brown*).

**Figure 2:**
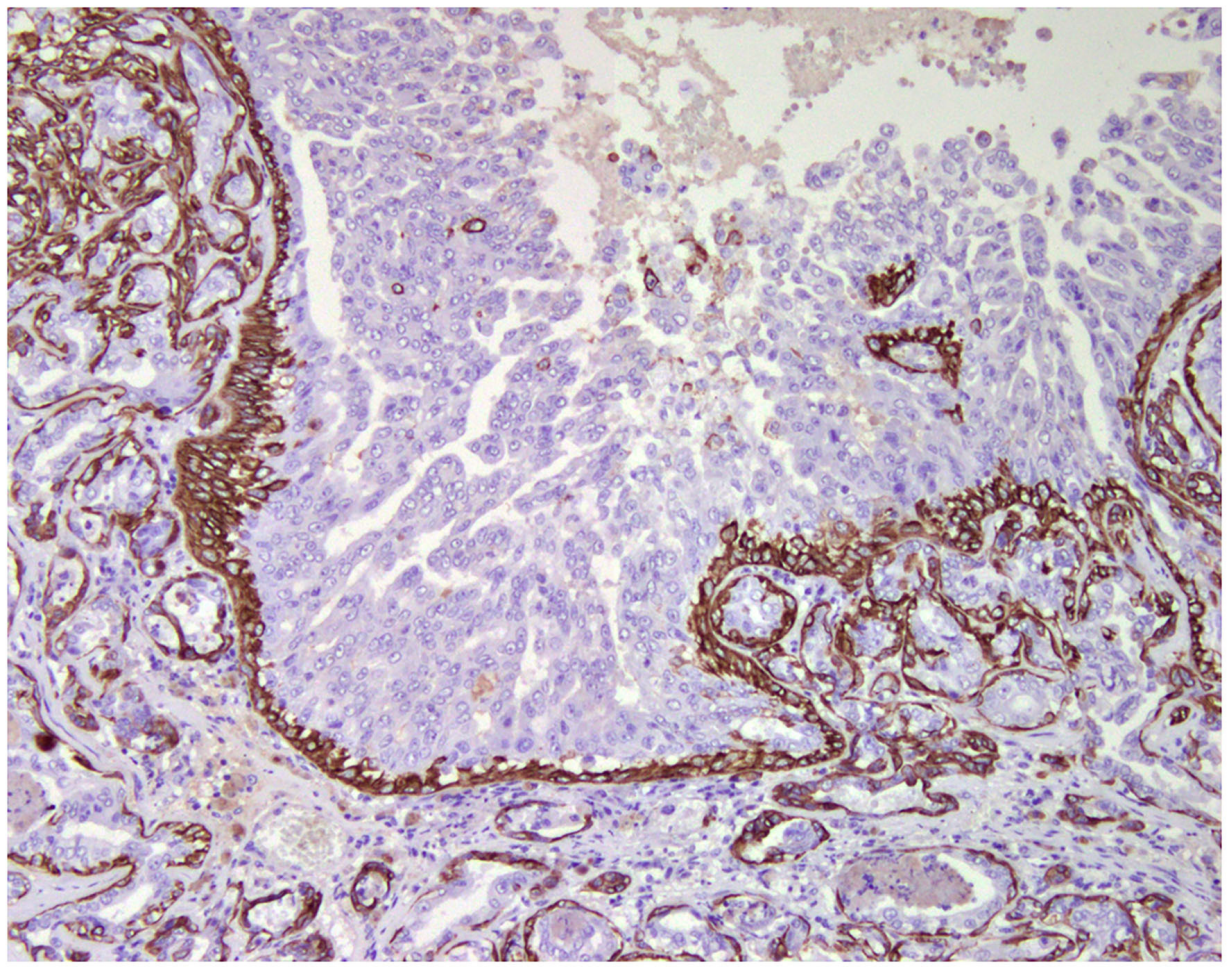
Tubulo-papillary carcinoma, mammary gland, dog. Immunohistochemical cytoplasmic staining of cytokeratin 14. Basal/myoepithelial cells are positive.

**Figure 3:**
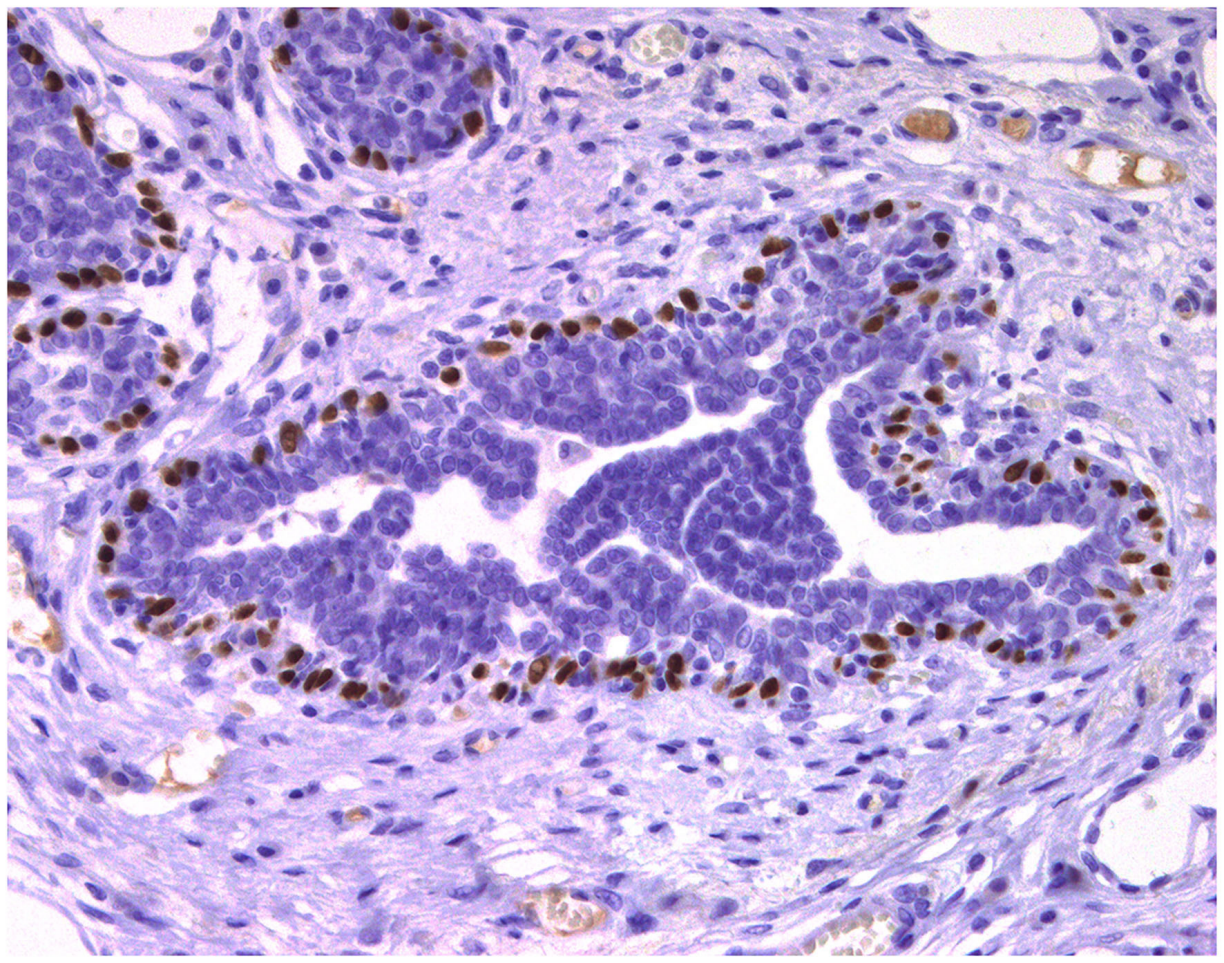
Epitheliosis, mammary gland, dog. Immunohistochemical nuclear staining of *p63*. Myoepithelial cells are positive.

**Figure 4:**
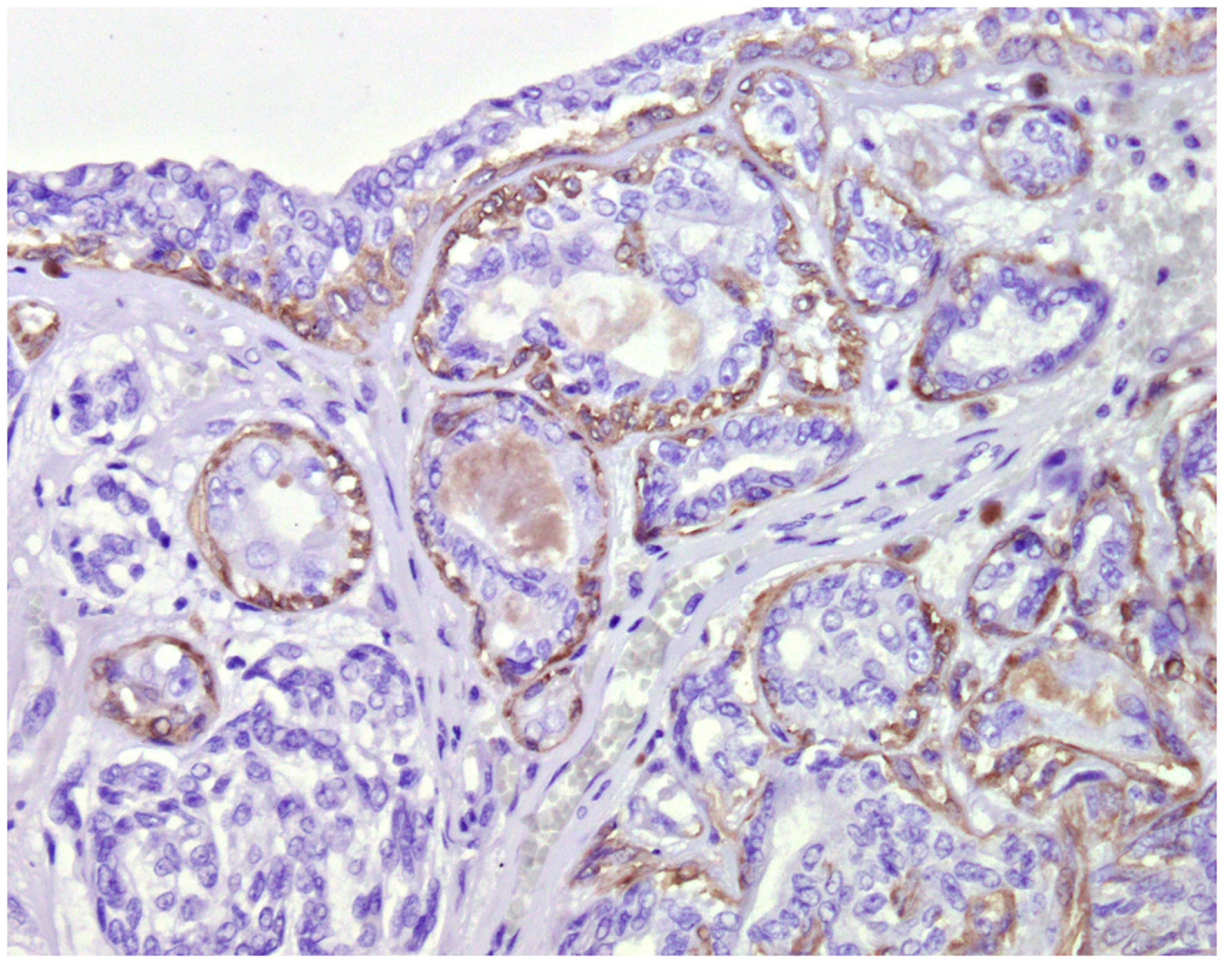
Tubular carcinoma, mammary gland, dog. Immunohistochemical cytoplasmic staining of calponin. Myoepithelial cells are positive.

**Figure 5:**
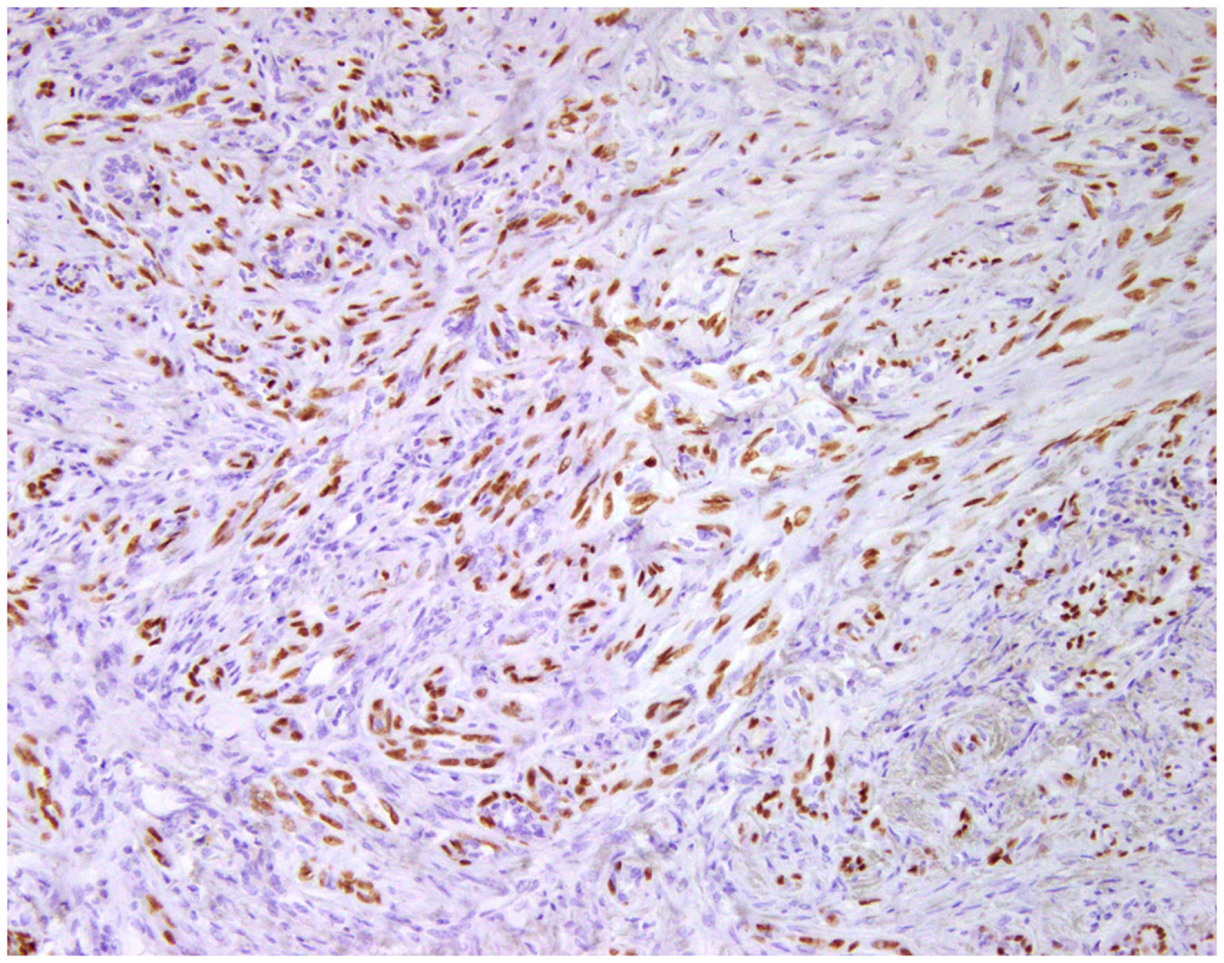
Carcinoma and malignant myoepithelioma, mammary gland, dog. Immunohistochemical nuclear staining of *p63*. Interstitial proliferated myoepithelial cells are positive.

Normal mammary gland cells of both humans and dogs have distinct immunoprofiles that can be used for diagnosis and potentially for targeted therapies. Luminal epithelial cells are characterized by the expression of low-molecular-weight (LMW) type I acidic cytokeratins (CKs) 18 and 19 and type II basic CKs 7 and 8. Basal/myoepithelial cells express high-molecular-weight (HMW) type I acidic CKs 14 and 17 and type II basic CKs 5 and 6 (Figures 1, 2). Myoepithelial cells, much more proliferative in CMC than in HBC (5), also express other markers such as p63, vimentin, calponin, smooth muscle actin (SMA), P-cadherin, CD10, epidermal growth factor receptor (EGFR), maspin, and 14-3-3 sigma protein (28–31) (Figures 3–5). However, a study performed in 2014 showed that, as happens in human breast (32), the mammary gland subpopulations are more complex than this. Using single and double immunohistochemistry (IHC) on serial sections of normal canine mammary gland, five distinct subpopulations were identified: (1) progenitor cells (CK5+, CK14+, p63+, and VIM+); (2) intermediary myoepithelial cells (CK5+, CK14+, p63+, SMA+, CALP+, and VIM+); (3) terminally differentiated myoepithelial cells (CALP+, SMA+, and VIM+); (4) intermediary luminal glandular cells (CK5+, CK14+, and CK8/CK18+); and (5) terminally differentiated luminal glandular cells (CK8/CK18+). The ducts are considered regenerative niches as they contain progenitor cells and intermediary luminal glandular cells; however, these are located in the basal position (33) and not in the luminal position, as occurs in women (34).

### Molecular Classification

In human breast pathology, IHC is routinely used to assist with the prognosis and to determine the specific treatment for patients (35). Over the past years, there has been considerable efforts to characterize and classify HBC at the molecular level to establish effective individual treatments. However, due to time and cost constraints, the surrogate molecular breast cancer classification is still largely based on IHC assessment of biomarkers: estrogen receptor (ER), progesterone receptor (PR), HER-2, and Ki-67, among others (36). Nowadays, HBC is classified on the following molecular subtypes by IHC (Table 1): luminal A (ER/PR+, HER-2–, and Ki-67 low), luminal B HER-2 negative (ER+, PR– or low, HER-2–, and Ki-67 high), luminal B HER-2 positive (ER+, PR+/–, HER-2+, and Ki-67 high), HER-2 enriched or overexpressed (ER/PR–, HER-2+, and Ki-67 high), and triple negative (ER–, PR–, HER-2–, and Ki-67 high). Triple-negative breast cancer (TNBC) can be further subdivided, according to genetic signatures, into luminal androgen receptor (LAR), mesenchymal (MES), and two basal-like subtypes (positive for high-molecular-weight cytokeratins): immunosuppressed (basal-like immunosuppressed, BLIS) and immune-activated (basal-like immune-activated, BLIA), depending on the upregulation or downregulation of genes associated with T, B, and natural killer (NK) cells (14, 36, 37).

**Table 1:** Classification of molecular subtypes in human breast cancer.

**Molecular subtypes**		**ER**	**PR**	**HER-2**	**Ki-67**	**HGM**
Luminal A		+++	+++	–	Low	Low
Luminal B HER-2 negative		+	+/–	–	High	Medium/high
Luminal B HER-2 positive		+	+/–	+/++	High	Medium/high
HER-2 enriched		–	–	+++	High	High
Triple-negative		–	–	–	High	High
LAR	AR+, MUC-1+					
MES	EMT genes+, PDGFRα+, c-Kit+					
BLIS	HMWCK+, VTCN1+					
BLIA	HMWCK+, STAT+					

*ER, estrogen receptor; PR, progesterone receptor; HER-2, human epidermal growth factor receptor 2; HGM, histological grade of malignancy; LAR, luminal androgen receptor; MES, mesenchymal; BLIS, basal-like immunosuppressed; BLIA, basal-like immune-activated; AR, androgen receptor; MUC-1, cell surface-associated mucin-1; EMT, epithelial–mesenchymal transition; PDGFRα, platelet-derived growth factor receptor α; c-Kit, stem cell factor receptor; HMWCK, high-molecular-weight cytokeratins; VTCN1, immunosuppressive V-set domain-containing T cell activation inhibitor 1; STAT, signal transducer and activator of transcription gene family*.

Despite the relevance of molecular subtyping in HBC, highly variable and even contradictory results have been obtained in CMC (10, 38–43). Regardless of the established guidelines for immunohistochemical assessment (31), variable application of the criteria has been utilized, and the percentages of the molecular subtypes differ enormously among investigations.

**Figure 6:**
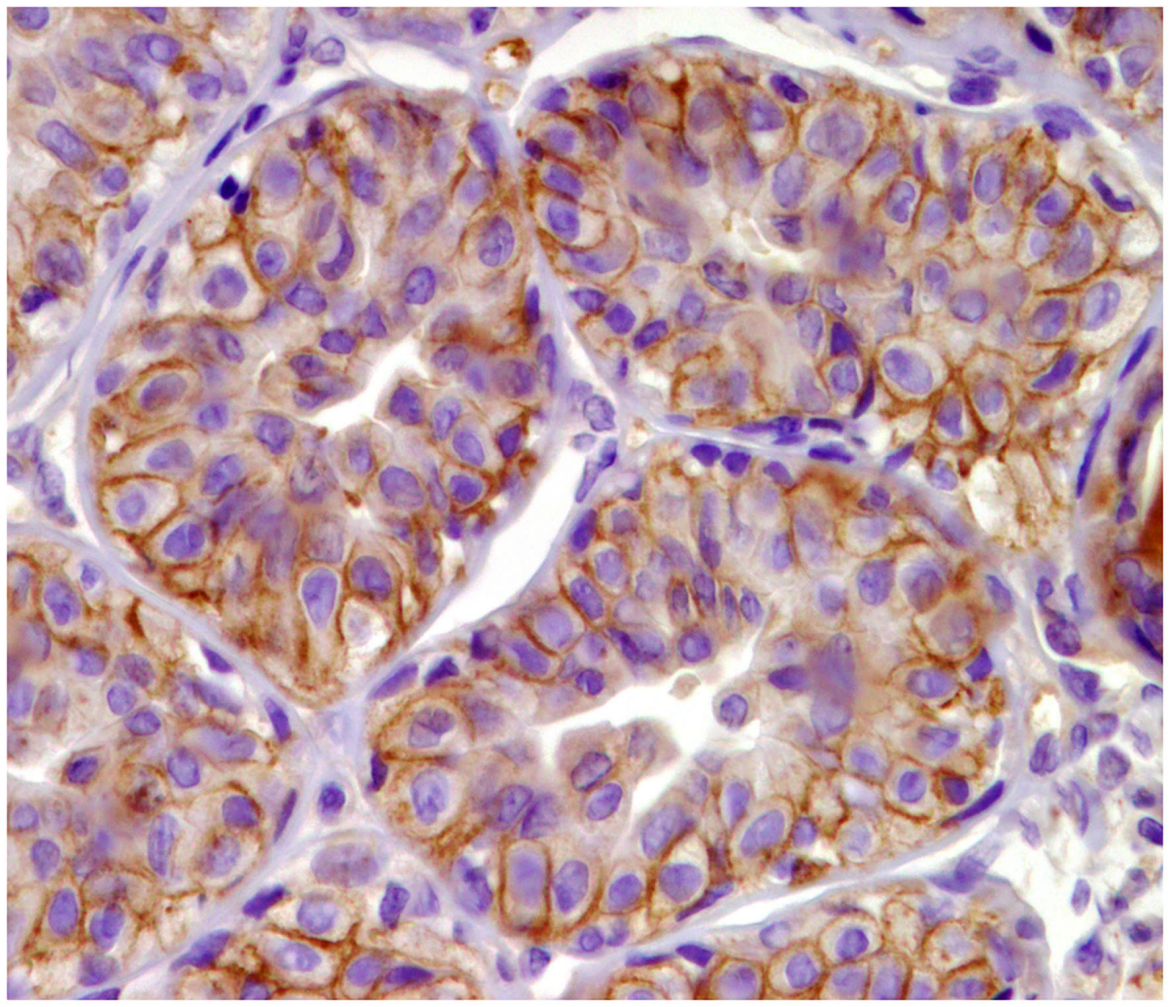
Tubular carcinoma, mammary gland, dog. Immunohistochemical membranous staining of human epidermal growth factor receptor 2 (HER-2). Complete and incomplete membranous staining of neoplastic cells.

HER-2 immunodetection in CMTs has always remained controversial. In a study performed by Abadie et al. (10), following appropriate standardized intrinsic and extrinsic controls (31), there were no HER-2-enriched tumors, in contrast to other studies in which between 5 and 15% of the mammary neoplasms were classified as HER-2 enriched (38, 42, 43). Previous studies identified HER-2 in normal, hyperplastic, and dysplastic mammary tissues and found no relation with prognostic parameters such as disease-free interval (DFI), overall survival (OS), and lymphovascular invasion, suggesting that HER-2 may play a role in the proliferation of mammary tissue in female dogs, but not conclusively in its malignant transformation (40). Furthermore, in a recent publication (44), HER-2 messenger RNA (mRNA) expression was observed in neoplastic and non-neoplastic mammary tissues using a novel quantitative RNA *in situ* hybridization assay, which correlates with the immunohistochemistry score. Among the non-neoplastic mammary tissues (hyperplasia), all cases showed HER-2: 21.4% were classified as 1+, while 78.6% were positive (2+ and 3+) (Figure 6). Moreover, within neoplastic tissues, no significant associations between HER-2 expression and clinical parameters were found.

The specificity of human anti-HER-2 antibody (Dako A0485) for HER-2 immunolabeling in canine tissues is also controversial. While one study showed no evidence of its specificity in canine tissues by Western blotting and subsequent mass spectrometric analysis (45), another work showed the cross-reactivity of the human anti-HER2 antibody in canine tissue (urothelial) by Western blotting (46).

Triple-negative tumors account for approximately half of CMCs (58.6%) (10), and showed significantly shorter disease-free interval (DFI) and overall survival (OS) in comparison to luminal A tumors. Comparable results were obtained in other studies: a triple-negative phenotype was related to a higher histological grade of malignancy, lymphatic invasion, and poorer prognosis. On the other hand, luminal A tumors were frequently complex tumors associated with better prognosis and longer DFI and OS (10, 38, 42, 43). In a study, HER-2-enriched and triple-negative CMCs presented a downregulation of E-cadherin compared to the luminal A and B subtypes, which are related to invasion and metastasis (43).

### Surgery

Surgery is the primary treatment in the control of CMTs; the goal is to remove the tumor(s) with clean margins and, depending on the case, to prevent the development of new tumors in the remaining glands (4). Clean margins have been found to be predictive of the median survival time (MST) in dogs with stages I–III (19), and very recent publications have elucidated new strategies for the intraoperative assessment of margins using near-infrared light waves to generate real-time, high-resolution images on the microscopic scale, similar to low-power histopathology (47–49).

Despite the elevated frequency of CMTs, there is a lack of prospective clinical trials robust enough to establish the extent of surgical excision: simple lumpectomy, local mastectomy, regional mastectomy, total chain mastectomy, or bilateral total mastectomy (4). Nevertheless, the current literature recommendations are the following: If a single, small (<1 cm) tumor is present, nodulectomy is usually carried out. Simple mastectomy is indicated when the tumor is larger and centrally located within the mammary gland. When multiple tumors are in consecutive glands, or a single tumor is found between two mammary glands, regional mastectomy (excision of adjacent mammary glands, from one to two or from three to five) is performed. Finally, total mastectomy is indicated when multiple tumors are distributed throughout the mammary chain, regardless of the size (4). Those cases in which surgery is not recommended are advanced metastatic (stage V) cancer (17, 50) and inflammatory mammary cancer (IMC) (7, 8, 51).

Additional treatment (adjuvant therapy) can be given after the primary mammary cancer treatment (surgery) to lower the risk of developing further recurrences and metastasis. Adjuvant therapy may include chemotherapy, radiotherapy, and targeted or individualized therapy, this latest based on the specific genetic characteristics of the cancer in a patient (52–55).

### Chemotherapy

Approximately 50% of the dogs with CMTs have at least a malignant neoplasm, and these patients would further profit from adjuvant chemotherapy. However, it has not been demonstrated conclusively if adjuvant chemotherapy offers a significant benefit to dogs with CMTs. Although cases have reported measurable tumor responses to doxorubicin (56–58), carboplatin (59, 60), mitoxantrone, and paclitaxel (61, 62), larger studies have not found a significant improvement of the measurable clinical responses (MST, DFI, or OS) using gemcitabine (17), doxorubicin, docetaxel (16, 19), and mitoxantrone (19). Due to the lack of efficient chemotherapeutics, dogs with malignant CMTs show high rates of recurrence (63) and poor prognosis (64).

Despite this uncertainty, chemotherapy is frequently used in those dogs with tumors considered at high risk of metastasis or recurrence (4).

Another chemotherapeutic approach is oral metronomic chemotherapy, which involves the administration of the lowest biologically effective dose at frequent regular intervals (65). In veterinary medicine, metronomic chemotherapy has been studied since 2007 (66) in tumors that include hemangiosarcoma (67–72), osteosarcoma (73–76), hepatic neuroendocrine carcinoma (77), primary lung carcinoma (78), soft tissue sarcomas (79–82), and transitional cell carcinomas (83), with chemotherapeutics such as cyclophosphamide, lomustine, and chlorambucil. To date, only one study has been published regarding CMC (84), in which longer MSTs were observed in patients treated with surgery and metronomic chemotherapy compared to dogs treated with surgery and conventional chemotherapy.

### Radiotherapy

Although radiotherapy is commonly used in HBCs with locoregional treatment (14), only one study has been published in CMC, specifically in dogs bearing IMC. Radiotherapy, in combination with piroxicam, toceranib, and thalidomide, showed significantly longer time to progression than those patients treated with the same regimen, but without radiotherapy (85).

## Adjuvant Targeted Therapies

Targeted therapies involve drugs that block the growth of cancer by interfering with individually expressed specific molecules responsible for tumor cell proliferation, survival, metastasis, or microenvironment (86). Given the poorly efficient available adjuvant therapies in dogs with mammary cancer, several studies have been made *in vitro* in animal models (mice) and few clinical trials, which are shown below.

### Hormonal Therapy

**Figure 7:**
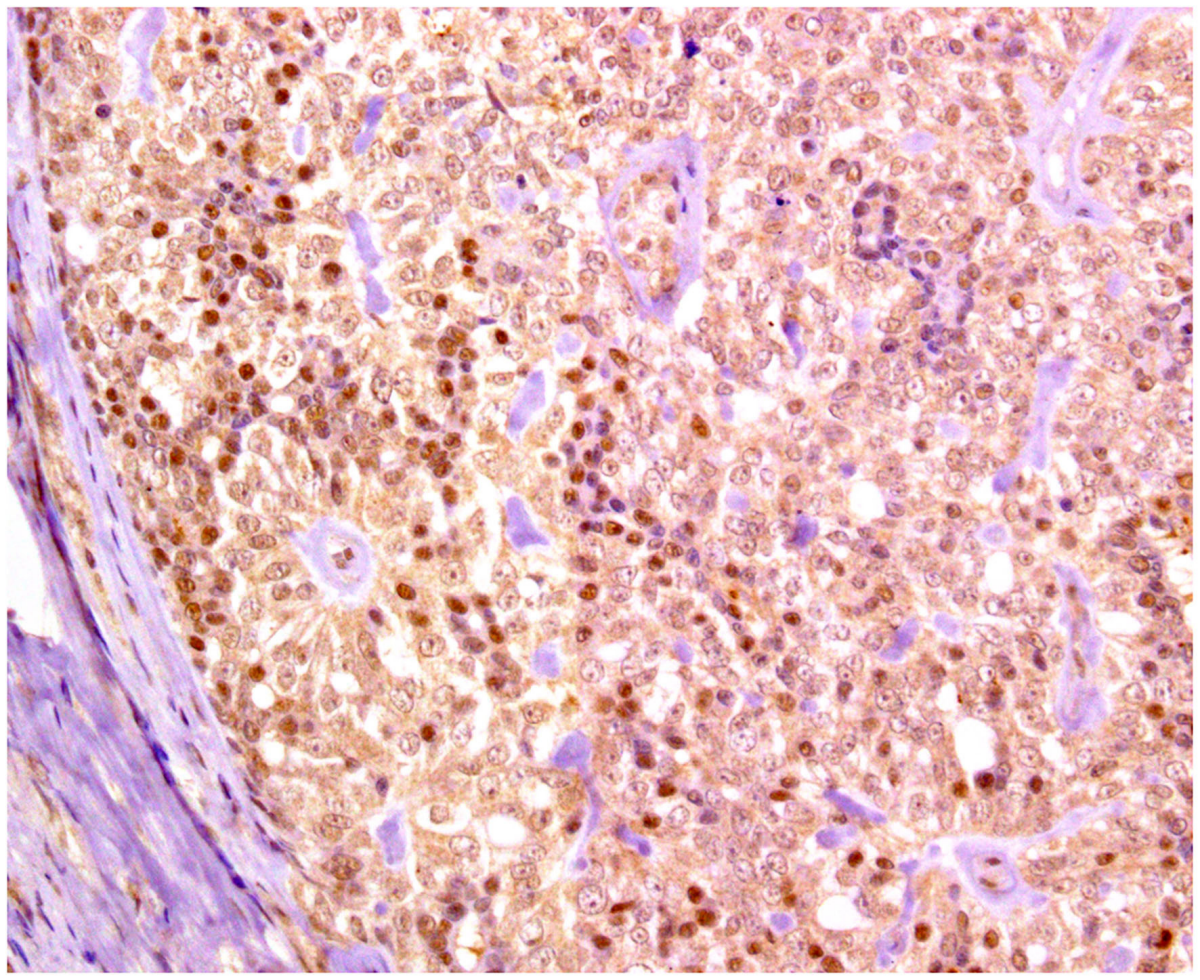
Solid carcinoma, mammary gland, dog. Immunohistochemical nuclear staining of estrogen receptor (ER). Neoplastic cells are positive with different intensities of immunolabeling.

**Figure 8:**
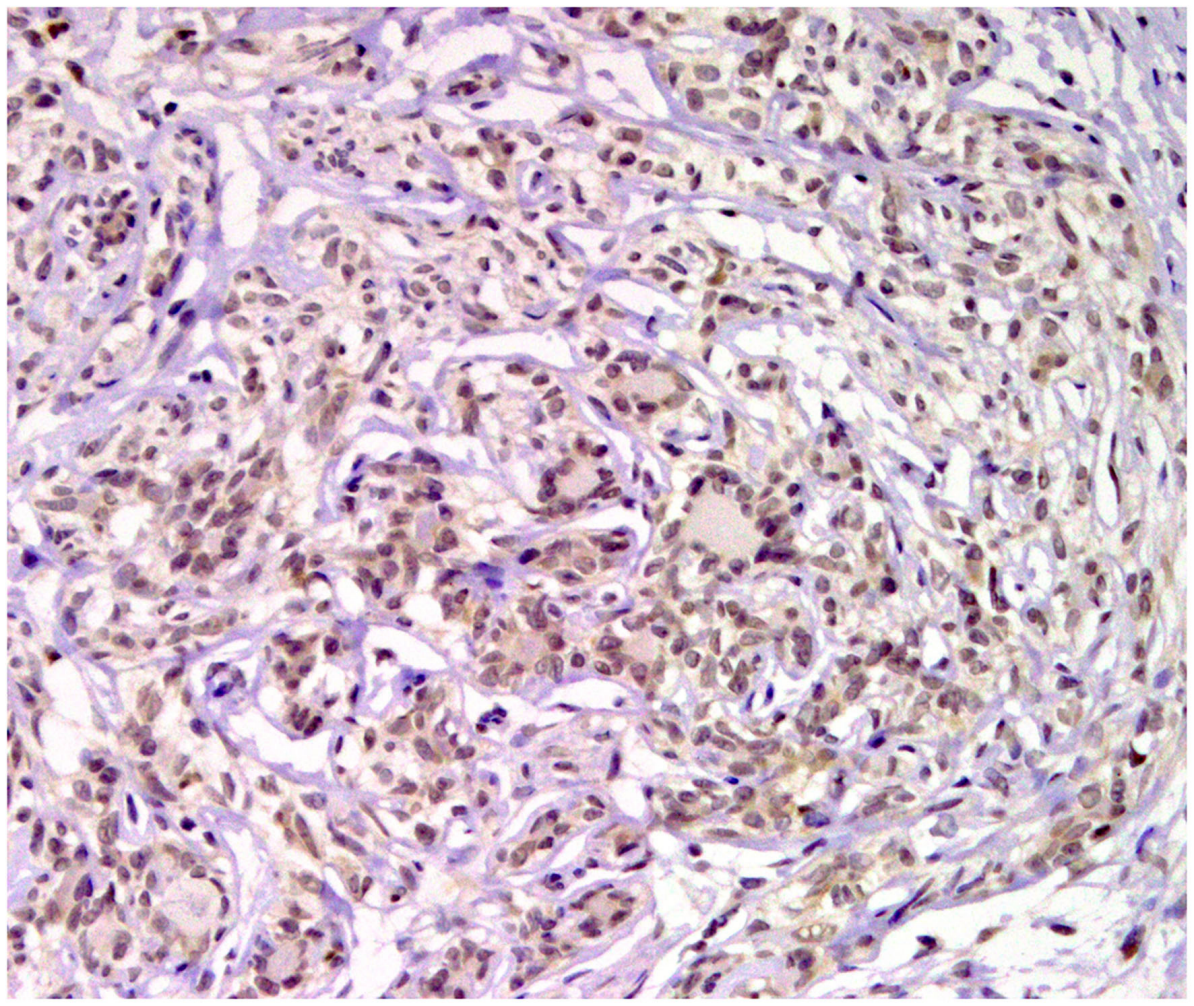
Tubular carcinoma, mammary gland, dog. Immunohistochemical nuclear staining of progesterone receptor (PR). Neoplastic cells are positive.

Estrogens, progesterone, prolactin (PRL), and growth hormone (GH) are essential for physiological mammary development. The effects of these hormones are mediated through binding to their respective receptors within the mammary gland (6) (Figures 7, 8). Estrogens and progesterone have been historically known to have a main role in tumorigenesis in CMTs, as spaying of female dogs before the first or second heat has significant protective effects (87). On the other hand, exposure to exogenous hormones (both estrogens and progestins) increases the risk of developing CMTs in dogs (88). Furthermore, studies have found that dogs with CMC have higher levels of estrogens in the blood, except in IMC, where the serum estrogens levels are lower than in dogs with other malignant mammary tumors (89, 90). On the other hand, ER, PR, PRL, PRL receptor, and GH receptor have been found to be downregulated in malignant mammary tumors compared to normal mammary gland (91, 92) and benign tumors (92–94). Furthermore, lymphatic invasion, high mitotic index, large tumor size, and tumor grade are significantly associated with low ER/PR expressions. Tumors with low ER/PR expressions have poorer prognosis (89, 93, 94).

In human medicine, ER+ breast cancer is the most common subgroup (>70%) (95). The risk of ER+ breast cancer increases with exposure to estrogens during a lifetime, for example due to an earlier menarche or late menopause (14). Moreover, hormone replacement therapy on menopausal women increases the risk of breast cancer (96). In women with HBC, ER+ tumors are susceptible to anti-hormone treatment. This therapy is designed to target mainly ER using antiestrogens, such as tamoxifen or fulvestrant, or by inhibiting the endogenous synthesis of 17β-estradiol using aromatase inhibitors (97).

Tamoxifen is a selective inhibitor of ER that is widely utilized in the treatment of HBC. However, in dogs, severe adverse effects (vulvar edema, vaginal purulent discharge, and pyometra) are repeatedly seen, and that outweighs the possible benefits of this hormone therapy (98, 99).

For this reason, other antiestrogens are being studied for their use in CMC. Indole-3-carbinol is a natural phytochemical found in cruciferous vegetables (i.e., cauliflower, cabbage, and broccoli) that has been proven to suppress cell proliferation and induce apoptosis in breast cancer cell lines by multiple mechanisms such as blocking estrogen receptors (100, 101). In veterinary medicine, a mouse xenograft model of canine IMC was treated with indole-3-carbinol, resulting in decreased tumor proliferation and increased apoptosis, although metastasis and lymphatic embolization were not prevented (102).

When women reach menopause, the ovaries no longer produce estrogen; however, this hormone is produced at other sites (fat, liver, muscle, and mammary tissue) through the aromatase enzyme (103). Aromatase mRNA levels are higher in cancerous tissues than in normal breast tissues in humans (104). Therefore, while ER inhibitors (e.g., tamoxifen) are preferentially given in premenopausal women with ER+ HBC, aromatase inhibitors are used for the treatment of postmenopausal patients with ER+ breast cancer (103). In dogs, IMC has been shown to express higher levels of aromatase than non-IMC. Moreover, *in vitro* treatment with letrozole, an aromatase inhibitor, significantly reduced cell proliferation in an IMC cell line (105). No clinical trials on the use of aromatase in dogs with CMC have been reported.

Melatonin is a hormone produced by the pineal gland in response to darkness, regulated by photoperiod (106). In breast tissue, melatonin exerts its action through two receptors: melatonin receptors 1 and 2 (MT1 and MT2, respectively) (107). A positive correlation between MT1 and ERα expressions has been demonstrated and recognized as a prognostic marker for OS in HBC (108). Furthermore, melatonin has been shown to suppress the proliferation of HBC cell lines *in vitro* and *in vivo* by disrupting estrogen-dependent signaling as well as inhibiting estrogen production in the gonads and in breast tissues through the aromatase pathway (109–111).

**Figure 9:**
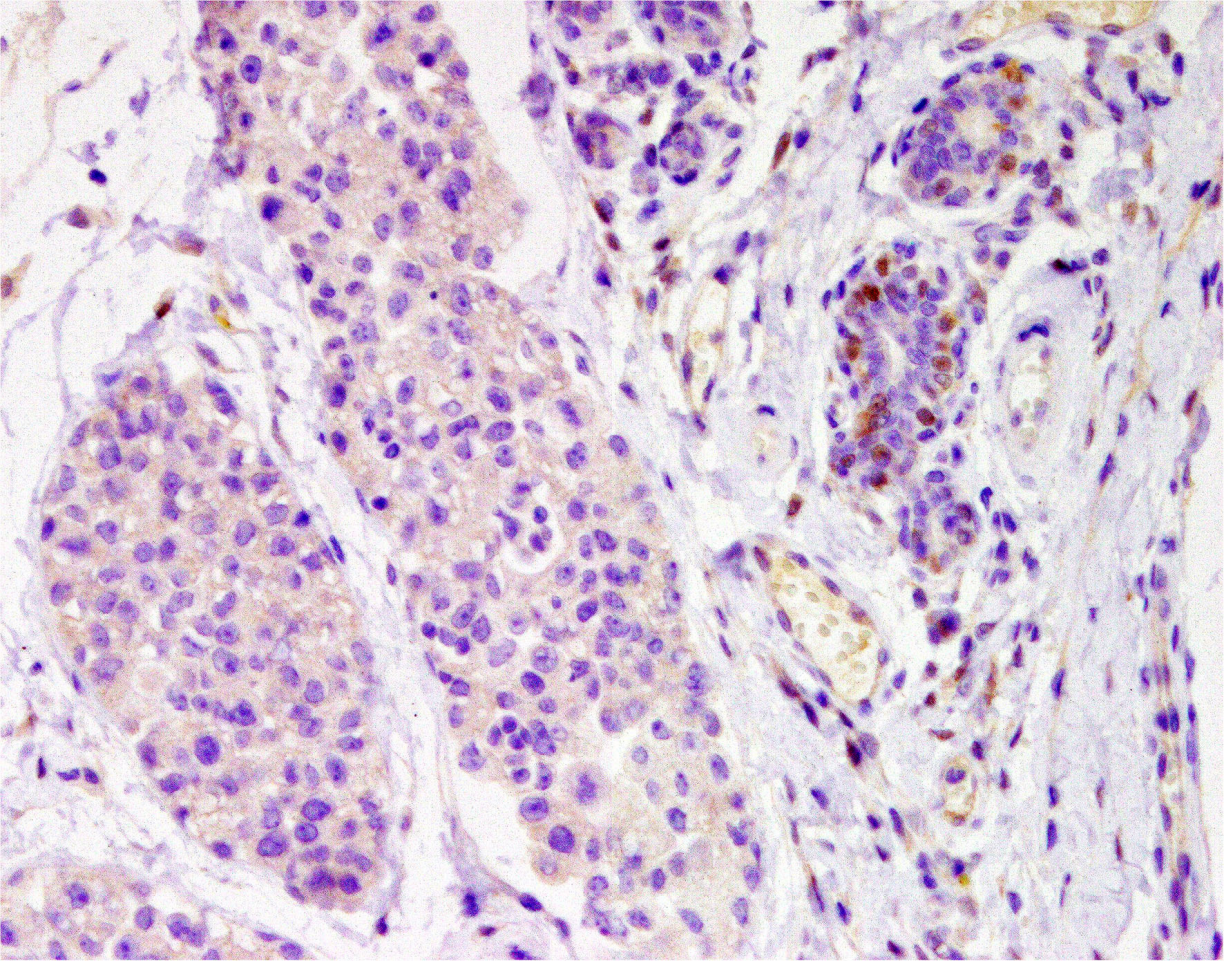
Tubular carcinoma, mammary gland, dog. Immunohistochemical nuclear staining of androgen receptor (AR). Neoplastic cells are negative; adjacent mammary hyperplastic cells are positive.

In the last decades, the role of androgens on HBC has started to gain the attention of researchers. Not only can androgens be a source of estrogen through the aromatase pathway but they have also been directly implicated as possible carcinogen factors for breast cancer (112). The androgen receptor (AR) is expressed in ~70–90% of invasive human breast cancers, a frequency comparable to or higher than those reported for ER (70–80%) and PR (50–70%) (113, 114). On the other hand, AR has been found in 64% of IMC and 40% of non-inflammatory CMC (89) (Figure 9). To date, in human medicine, AR-targeted drugs have been approved for the treatment of prostate cancer, and different AR inhibitors are being investigated for the treatment of HBC, specifically for the LAR subtype of triple-negative breast cancer (115). Flutamide is an analog of androgen that blocks the AR (116). It is already used in veterinary medicine to treat canine prostate hyperplasia and cancer (117), but has not been tested in patients with CMC. Flutamide has been shown *in vitro* to decrease the proliferation of an IMC cell line, and reductions in the tumor size and metastasis rates in IMC xenografted mice were found (118).

Progesterone has also a relevant role in HBC (119) and in CMC (105, 120). Progesterone signals *via* PR, whose expression is stimulated by estrogen (121). Some authors have suggested that progesterone may lead the transition of tumors from luminal to a basal phenotype (122). Upon progesterone exposure, luminal cells secrete growth factors (RANKL and Wnt) that may stimulate the recruitment and differentiation of cancer stem cells (CSCs, characterized by CD40^HIGH^ and CD24^LOW^) (123).

Therapeutic targeting of PRs have been studied in HBC as a treatment of endocrine refractory breast cancer, the most common drugs being megestrol acetate, medroxyprogesterone acetate, mifepristone, and onaprestone (124). The antiprogestin aglepristone is employed in veterinary medicine for abortion, parturition induction, and pyometra treatment in female dogs (125). The study of antiprogestins in oncology has begun relatively recently. Mifepristone and onapristone have been shown to decrease the number of viable tumor cells *in vitro* in a canine mammary carcinoma cell line (126). *In vivo*, aglepristone treatment diminished the expression of PR, reduced the proliferation index in PR+ CMTs (127), and significantly increased DFI and OS in cases with PR+, <3 cm, low and medium grade, low proliferative tumors (128). Although promising for PR+ CMTs, antiprogestins in veterinary oncology need further studies with larger series and longer follow-up periods.

Oxytocin is a peptide hormone mainly synthesized in the hypothalamus that plays a role in uterine contraction and milk ejection, among other functions, and has been linked to the mammary carcinogenic process. Human (129) and canine (130) carcinoma cell lines and HBC xenografted mice (131) have shown reduced proliferation after oxytocin treatment. Recently, it has been shown that the expression of oxytocin receptors in CMTs is associated with ER+, benign tumors, and low-grade malignant tumors compared to high-grade malignant tumors (132).

Desmopressin is a synthetic analogous of vasopressin (antidiuretic hormone) that binds to the V2 membrane receptor (V2R); it has been used for the management of diabetes insipidus in humans (133) and dogs (134). Since V2R is also expressed in endothelial cells, desmopressin has been employed in the treatment of different bleeding disorders due to its effects in the hemostatic system (135). In oncology, a number of studies have shown in mouse models of HBC and in different HBC cell lines that this peptide seems to have anti-metastatic and anti-proliferative effects, probably by targeting V2R-expressing cancer cells and raising intracellular cAMP (136, 137). In a study on canine mammary carcinoma cell lines, desmopressin was shown to decrease cell viability at high concentrations (130). Furthermore, a veterinary clinical trial has demonstrated that the perioperative administration of desmopressin increases the DFI and OS in CMC (138). Although it seemed a very promising therapy, there have been no subsequent clinical trials in veterinary or human medicine performed by this group, with the exception of a phase II trial in HBC patients in 2015 (139), where the safety of perioperative administration was established; however, the effect on DFI, OS, or any other clinical parameter has not been reported. Due to this controversy, Sorenmo et al. conducted a prospective randomized trial in dogs with CMC, in which no metastasis-preventing effect of desmopressin was found (140).

### Tyrosine Kinase Receptors

Tyrosine kinase receptors (TKRs) catalyze a series of phosphorylation of target proteins that play a significant role in cell proliferation, metabolism, motility, survival, and apoptosis, as well as endothelial cell activation, leading to neovascularization.

Breakthroughs in biotechnology over the past decades have led to the development of new molecules that act on specific targets, among which small-molecule tyrosine kinase inhibitors (TKIs) and monoclonal antibodies (mAbs) stand out. Small-molecule (below 900 Da) tyrosine kinase inhibitors rapidly diffuse across cell membranes and target intracellular or extracellular proteins (kinases). To identify TKIs, the suffix “nib” is placed at the end of the generic name (141). On the other hand, monoclonal antibodies cannot cross cell membranes, therefore acting on the extracellular targets. The stem “mab” at the end of the name corresponds to mAbs. When the monoclonal antibody is completely human, the “umab” substem is used (e.g., nivolumab). If the immunoglobulin is chimeric (human constant domain, plus non-human variable domain), the mAb is named with the “ximab” substem; when the antibody is humanized (human framework with grafted murine complementary determining regions), then the “zumab” substem is utilized (e.g., trastuzumab) (142).

Among TKRs, HER-2, vascular endothelial growth factor receptors (VEGFRs), platelet-derived growth factor receptors (PDGFRs), stem cell factor receptor (c-KitR), and colony-stimulating factor 1 (CSF-1) are overexpressed or constitutively activated in human and canine tumors (143, 144). Tyrosine kinase inhibitors act by competitive inhibition of ATP binding, thus avoiding consecutive phosphorylation reactions and blocking signal transduction to the nucleus, inducing the deregulation of cellular proliferation and differentiation (145). Most of the TKIs are given orally, which means a huge benefit for animal welfare, diminishing stressful situations and providing ease of administration by the owner (146). For these reasons, several attempts to block these receptors have been made in veterinary oncology. Some TKIs designed for human oncology can have multiple actions depending on the receptor blocked (i.e., sunitinib inhibits VEGFR, PDGFR, c-KitR, and CSF-2) (147) and can be served for different types of cancer. Below, we review these targets separately.

#### Anti-Her-2

The family of EGFRs encompasses four tyrosine kinases receptors also named human epidermal receptors (HERs): HER-1, HER-2, HER-3, and HER-4. When activated, these receptors trigger numerous signaling pathways, which regulate cell proliferation and survival, as well as the metastasis of tumor cells (144). Approximately 15–25% of HBC show overexpression of the HER-2 protein and/or amplification of the *HER-2* gene, which is generally associated with a poor prognosis and an aggressive disease course (148).

The targeted therapy with anti-HER-2 agents in HBC is well-established. Several classes of anti-HER-2 agents have been developed, including: (1) monoclonal antibodies that bind to the extracellular domain of HER-2, such as trastuzumab and pertuzumab, which act by direct inhibition of HER-2 and indirect activation of the immune system to evoke antibody-dependent cellular toxicity; (2) small-molecule TKIs, including lapatinib, neratinib, and afatinib, that bind to the intracellular tyrosine kinase domains of HER-2 and other HER family members; and (3) antibody–drug conjugates, e.g., trastuzumab emtansine (T-DM1), composed of a monoclonal antibody directed at the extracellular domain of HER-2 linked to a cytotoxic agent. All of the latter have been approved by the European Medicines Agency (EMA) for clinical use in patients with HER-2+ HBC (149).

The amino acid homology values between canine and human EGFR-1 and HER-2 are reported to be 91 and 92%, respectively (150). *In silico* studies (research conducted by computer modeling or simulation) have shown that cetuximab (monoclonal antibody against EGFR-1) epitopes only differ by four amino acids in canines, while the trastuzumab (monoclonal antibody against HER-2) binding site is identical in humans and canines. *In vitro* studies with canine mammary carcinoma cells have reported a significant growth inhibition and G^0^/G^1^ phase arrest when treated with either cetuximab or trastuzumab (150). A “caninized” version of cetuximab developed by the same group, fusing the canine constant heavy-chain genes with the variable region murine genes of cetuximab, was able to inhibit the proliferation of canine mammary carcinoma cell lines, enhancing tumor cell killing *via* (151) phagocytosis.

With regard to anti-HER-2 TKIs, only gefitinib has been attested in CMTs. *In vitro* studies showed anti-proliferative effects in a canine mammary carcinoma cell line comparable to those with small interfering RNA (siRNA) targeting EGFR and HER-2 (152).

There are no clinical trials published on the use of HER-2 inhibitors in CMC, and their potential use in veterinary medicine is still far from daily routine.

#### Anti-angiogenesis

Since 1971, it is well-known that the growth of solid tumors, beyond the size of 1–2 mm^3^, is conditioned to a sufficient supply of nutrients and oxygen, for which the new development of blood vessels was hypothesized and called angiogenesis (153). In early tumor development, neoplastic cells are oxygenated through simple diffusion in a phase defined as “avascular state.” With time and growth, tumor cells are deprived of oxygen and undergo a phenotypical change into a pro-angiogenic state (angiogenic switch) by inducing specific gene expression to overcome hypoxia through sprouting angiogenesis, vasculogenic mimicry (VM), and/or vascular co-option (VCO) (154). The sprouting angiogenic process occurs in both normal and neoplastic tissues. It is a complex process regulated by pro- and anti-angiogenic factors, among which stands out the VEGF family receptors, especially VEGF-A and its receptor (VEGFR-2), the fibroblast growth factor receptor 2 (FGFR-2), and PDGFR (154). VM and VCO are only found in highly aggressive cancers, as is explained below.

In both, humans (155) and dogs (156), VEGFR-2 and PDGFR are increased in malignant, triple-negative mammary tumors, being higher in those cases with metastatic disease (distant > regional) and positively correlated with tumor grade. Further, in HBC, microvascular density (MVD) is significantly correlated with metastasis, OS, and DFI (157); likewise, MVD is increased in canine primary mammary tumors with distant metastasis (156, 158).

In human medicine, about one third of the molecular therapeutics in clinical development are directed against angiogenesis. Angiogenesis is mediated by two major molecular routes: the VEGF axis-dependent route and the non-VEGF-mediated mechanisms (159). The anti-angiogenic therapies against the VEGF family block either the ligands or the receptors (160). The most widely studied anti-angiogenic therapeutic is bevacizumab (monoclonal antibody against the anti-VEGF-A ligand). In 2008, it became the first Food and Drug Administration (FDA)-approved anti-angiogenic drug for HBC; however, due to the lack of significant clinical improvements in subsequent studies, the approval was revoked in 2011 (161).

Small-molecule TKIs that block the VEGF family receptors have also been developed (pazopanib, sunitinib, and sorafenib, among others), many of them not only acting against angiogenesis but also diminishing other tumor metabolic pathways (162). For instance, pazopanib acts as an anti-angiogenic through the inhibition of VEGFR, PDGFR, and c-Kit in human renal carcinoma, soft tissue sarcoma, and breast cancer in combination with the anti-HER-2 lapatinib (163). In addition, sunitinib, which inhibits VEGFR, PDGFR, c-KitR, and CSF-1R, has shown promising activity as a single agent for advanced HBC (164). On the other hand, sorafenib, a VEGFR, PDGFR, and rapid accelerated fibrosarcoma-1 (Raf-1) kinase inhibitor, has antitumor effects *in vitro* and inhibits neovascularization in xenograft models of HBC (165). Sorafenib has been assessed in veterinary medicine, and it has shown a promising ability to inhibit VM in CMC cell lines *in vitro* (166). Other TKIs that target VEGFR-2 and that have been proven to significantly diminish the level of active (phosphorylated) VEGFR2, reduce cell proliferation and migration, and increase apoptosis in *in vitro* studies against CMT cell lines are rivoceranib (apatinib) (167) and vandetanib (168).

Despite the logical targeting of angiogenic pathways in cancer treatment and significant efforts in new drug development and HBC clinical trials, no significant clinical benefit has been achieved that outweighs the potential side effects. Some authors have hypothesized that a multi-target approach to angiogenesis is needed to overcome the apparent resistance of tumors to anti-angiogenesis treatment (159).

As stated earlier, angiogenesis is not the exclusive method to nourish tumor cells; two other mechanisms have been discovered in highly aggressive neoplasms—VM (169, 170) and VCO (171)—which have been hypothesized as responsible for the resistance to anti-angiogenic therapy (172–174). VM describes the formation of *de novo* vascular channels lined by genetically deregulated highly malignant cancer cells (175). These cancer cells, also called endothelial-like cells, exhibit cancer stem cell markers and characteristic endothelial morphology under electron microscopy in cell lines of human and canine inflammatory mammary cancer (176). VM has been associated with the spread and metastasis of human (177) and canine mammary tumors (178). VM was found in 33% of canine mammary tumors, and its presence was correlated with histological grade of malignancy and shorter survival times (166).

In VCO, neoplastic cells closely adhere to preexisting blood vessels to obtain nutrients and oxygen and further develop sprouting angiogenesis after the hypoxia switch is turned on (174, 179). In veterinary medicine, VCO has not been recognized yet.

#### Anti c-Kit and Other Receptors

**Figure 10:**
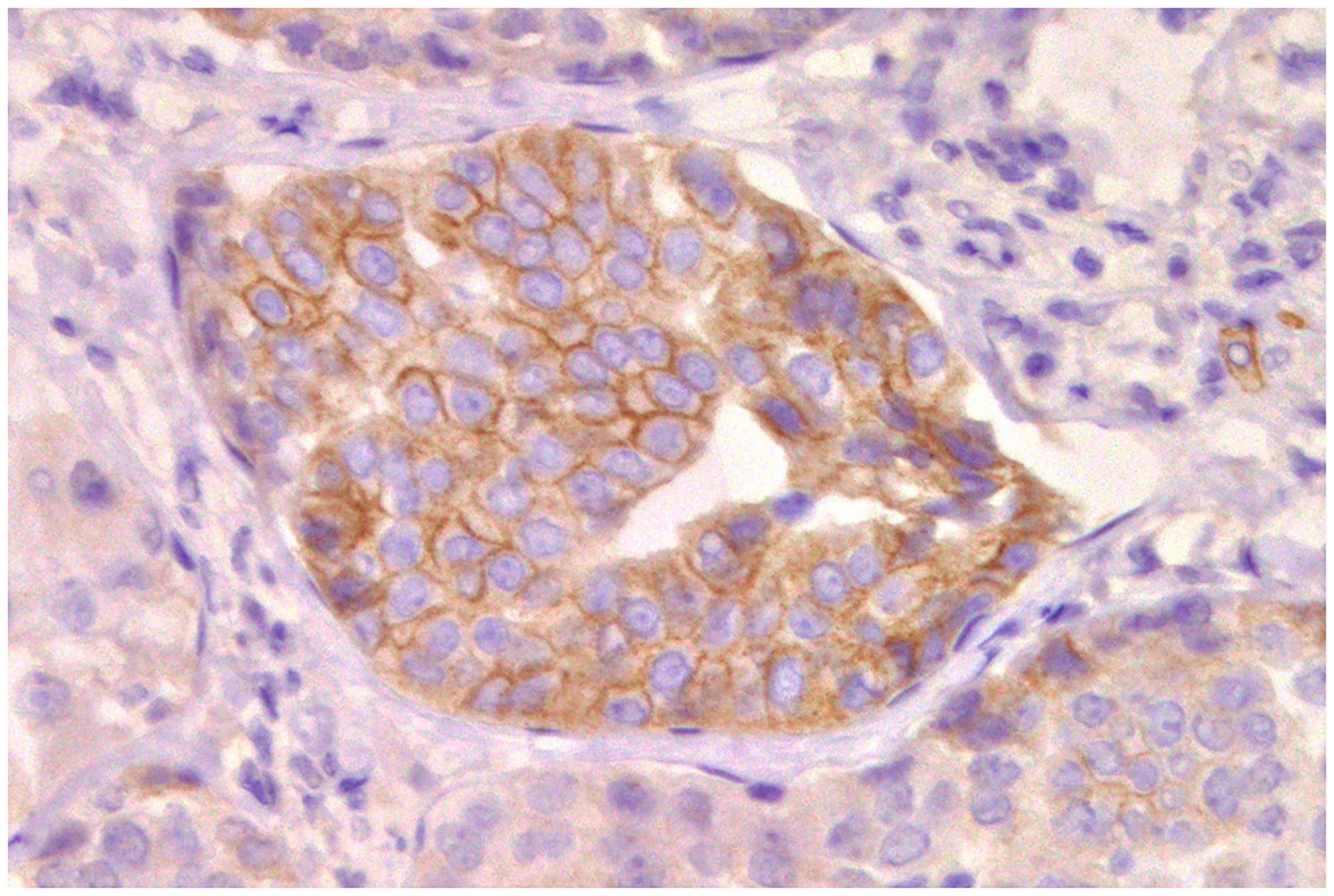
Tubular carcinoma, mammary gland, dog. Immunohistochemical membranous staining of stem cell factor receptor (c-KitR). Neoplastic cells are positive.

The stem cell factor receptor c-Kit is an active participant in many vital functions in humans and animals, such as homeostasis, cell maintenance, differentiation, and melanogenesis, in a wide variety of cells (180). However, overexpression or mutation where the receptor is constitutively activated has been detected in a number of tumors in humans (181) and dogs, notably in canine mast cell tumors (143). In canine mast cell tumors, the c-Kit receptor can be labeled by immunohistochemistry in three different staining patterns: pattern I (perimembranous staining), pattern II (focal or stippled cytoplasmic), and pattern III (diffuse cytoplasmic staining), pattern III being the more aggressive (182). In women, c-Kit is expressed in normal breast tissue and is gradually lost during the malignant progression of breast tumors due to a downregulation at the mRNA level (183). On the contrary, c-Kit has been found to be present in 38.5% of CMTs (184) (Figure 10) and seems to be overexpressed in malignant mammary tumors (185, 186), in addition to being correlated with the proliferation index (187) and angiogenesis (188).

In veterinary medicine, there are only two approved targeted anti-c-Kit TKIs: toceranib (Palladia) and masitinib. Toceranib, designed and approved for mast cell tumors in dogs, acts mostly through the inhibition of the c-KitR, and it is a sister compound to sunitinib, which was later approved for human therapies (189). Toceranib appears to exert antitumor activity against a variety of dog cancers, including CMC, particularly in cases with pulmonary metastases (143). Toceranib also has anti-angiogenic effects, inhibiting VEGFR and PDGFR, and produces a significant decrease in regulatory T cells, which may increase immune surveillance (189). Toceranib has shown a discrete *in vitro* reduction in cell proliferation on canine mammary carcinoma cell lines (184).

The evaluation of toceranib in clinical trials with dogs with CMC is very limited. In a study with dogs presenting IMC, toceranib was given in combination with piroxicam (anti-COX-2) and thalidomide (an immunomodulatory and anti-angiogenic agent) (190), with or without hypofractionated radiation therapy. The authors found a significant improvement in the clinical benefit rate and overall survival time compared with historical palliative treatment. The response was better when radiotherapy was employed (85).

Masitinib is also a potent and selective inhibitor of the c-Kit receptor that has been recommended for its use in canine mast cell tumors (191). It also inhibits the PDGF receptor and the fibroblast growth factor receptor (FGFR-3). Masitinib has been studied *in vitro* as a “chemosensitizer” by enhancing the anti-proliferative effects of a cytotoxic drug, gemcitabine (192). There are no other studies on the treatment of CMC.

Although TKIs (in particular toceranib) are commonly used for CMC treatment, in the veterinary clinic, the evidence is merely anecdotal, and only the aforementioned studies, one *in vitro* (184) and two *in vivo* (85, 189), have been conducted. There is an urgent need for prospective randomized studies to adequately evaluate the effectiveness of toceranib in patients with CMC in c-Kit-positive or c-Kit-negative tumors.

Other tyrosine kinase inhibitors are under study. Palbociclib is an inhibitor of cyclin-dependent kinase 4 (CDK4) and CDK6, which are key regulators of the cell cycle machinery and, thus, cell proliferation (193). In women, palbociclib improves progression-free survival in ER+, HER-2– breast cancer when combined with an aromatase inhibitor (letrozole) or an ER downregulator (fulvestrant), so it received approval from the FDA and EMA (194). CDK6 has been consistently detected in CMT cells with no association to histotype or grade (64). *In vitro* studies with canine mammary cell lines have shown that palbociclib induces cell cycle arrest, prevents colony formation, and impairs cell migration activity (64). There are no clinical trials on dogs with CMC.

### Antitumor Suppressor Gene *p53*

The tumor suppressor gene *p53* plays a central role in tumorigenesis. The p53 protein, after tetramerization, inhibits cell proliferation, functioning as an initiator of cell cycle arrest and apoptosis. Mutations of *p53* often cause a disruption of its tumor-suppressor function and induce genomic instabilities (195). Mutations in the *p53* gene are associated with more than half of all human cancers and have been described in multiple cancers in dogs (196), including mammary tumors (197, 198). However, its expression and mutation status as prognostic factors in veterinary medicine are controversial: while some studies have found no correlation (199, 200), other authors have associated higher levels of *p*53 with poor overall survival (201, 202).

Mutation of the *p*53 gene can lead to a stable protein that is identifiable by immunohistochemistry (203), although a truncating mutation of the *p*53 gene can lead to an immunohistochemically undetectable protein (204). Therefore, four different patterns of *p*53 immunolabeling have been published: overexpression, complete absence, cytoplasmic staining, and wild-type staining. The first three patterns are related to an underlying *p*53 mutation, while the fourth pattern is primarily associated with no mutation (204, 205). Altered expressions of *p*53 may result from a direct mutation of *p*53 within tumor cells or from an altered localization of *p*53 by increased export of the protein from the nucleus, thereby decreasing its downstream targets and inhibiting apoptosis and cell cycle arrest (195).

Approximately 30% of HBCs have a *p53* mutation, but this frequency is dependent on the molecular subtype, as the luminal subgroup has the lowest mutation rate and TNBC has the highest (up to 88%) (206). Additionally, *p53* overexpression has been correlated with more aggressive HBCs and worst outcomes (207–209).

In CMC, malignant tumors have shown higher levels of *p*53 than benign tumors (210), and the increase of *p*53 is greater in higher-grade tumors with higher proliferation rates (211). Likewise, a significant correlation between increases in *p*53 expression and mutations with shorter OS has been found (212).

Since mutations in the *p*53 gene have an enormous impact on cancer development, great efforts are being made into the mechanisms that can counteract this effect and are brought together in several extensive reviews (213, 214) that compile different experimental approaches to target *p*53 in human cancer: inhibition of mutant *p*53 by promoting its protein degradation, restoration of the wildlife activity of mutant *p*53, and immune stimulation against *p*53 activity.

In contrast, there is only one study on p53 therapy in canine mammary cancer cells. Neoplastic cells can increase the cytoplasmic translocation of nuclear p53 by overexpressing exportin-1, thus preventing its binding to DNA and its anti-proliferative activities (215). The addition of KPT-185 and KPT-355, engineered molecules that inhibit exportin-1 on canine mammary carcinoma cells, *in vitro* induced cell cycle arrest, apoptosis, and reduced growth (216).

### Anti-cyclooxygenases

Cyclooxygenases (COX) are a group of enzymes that catalyzes the conversion of arachidonic acid to prostanoids (prostaglandins, prostacyclins, and thromboxanes). In humans and animals, COX exist in three isoforms: COX-1, constitutively found in most cells, in charge of maintaining homeostasis, protection of the gastric mucosa, and regulation of platelet aggregation and renal blood flow; cyclooxygenase-2 (COX-2), an inducible isoform that is detected in neoplastic and normal cells induced through several stimuli (e.g., mitogens, growth factors, hormones, and pro-inflammatory cytokines); and COX-3, which is expressed mainly in the central nervous system and the aortic wall (217, 218).

Since 1897, with the development of aspirin, non-steroidal anti-inflammatory drugs (NSAIDs) have been developed as analgesic, antipyretic, anti-inflammatory, and anti-rheumatic treatment (219). However, a meta-analysis showed a remarkable preventive effect against colon cancer (220). Other studies found the same effects in different types of cancer, such as breast cancer (221). Nevertheless, the adverse effects of long-term use of NSAIDs were significant, mainly gastrointestinal bleeding, increased uric acid, and coagulation inhibition, among others (222). In an attempt to avoid these adverse effects and target specifically COX-2 for its participation in the neoplastic process, selective COX-2 inhibitors were designed and named as “coxibs.” Experimentally, both NSAIDs and coxibs have been shown to inhibit tumorigenesis by inhibiting cancer cell growth and proliferation, modulating apoptotic activity, reducing the metastatic and invasive potential of cells, and by inhibiting angiogenesis (223, 224).

Coxibs had been related in human medicine to thrombotic cardiovascular events, myocardial infarction, and stroke, especially in long-term use (225). Although subsequent meta-analysis revealed that the risk of cardiovascular events was not related to the usage of coxibs (226), the use of coxibs as adjuvants in the treatment of human cancer is an ongoing intense field of research, especially in combination with chemotherapeutic agents (227).

COX-2 is overexpressed in various canine epithelial malignant tumors, including ovarian carcinomas, prostate carcinomas, urothelial and transitional cell carcinomas, colorectal and small intestine tumors, squamous cell carcinomas, osteosarcoma, and melanoma (228).

**Figure 11:**
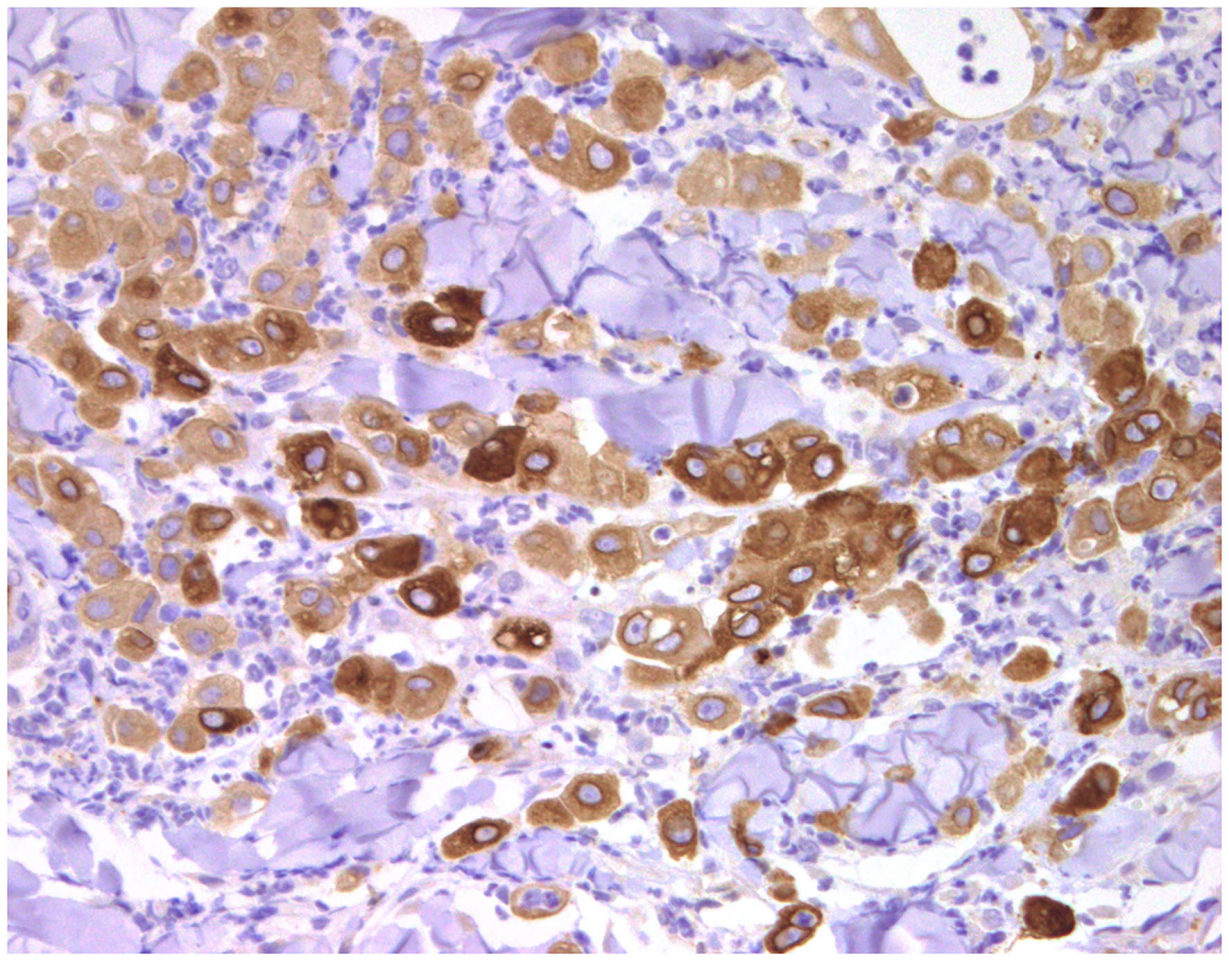
Anaplastic carcinoma, mammary gland, dog, inflammatory carcinoma. Immunohistochemical cytoplasmic staining of cyclooxygenase-2 (COX-2). Neoplastic cells are positive with different intensities of immunolabeling.

Since 2003, COX-2 expression has been reported in both benign and malignant canine mammary tumors (229) (Figure 11). Cyclooxygenase-2 is overexpressed in 83–95% of the CMC in association with characteristics of aggressiveness, such as high histological and nuclear grades, mitotic index, and lymph node metastasis (230–232). COX-2 has an important role in the angiogenesis of CMC: its presence is correlated with MVD and EGFR and VEGF expressions (233). Interestingly, in canine IMC, which is characterized by exacerbated angiogenesis, lymphangiogenesis, and lymphangiotropism, COX-2 is associated with higher lymphatic proliferation index, VEGF-D (a lymphangiogenic factor), and its receptor VEGFR-3; in contrast, COX-2 is associated with VEGF-A in non-inflammatory mammary cancer (234), indicating a different role of COX-2 in the angiogenesis of IMC.

Moreover, COX-2 also participates in the immunomodulation of the tumor microenvironment by promoting the M2 phenotype of macrophages, inhibiting antigen-presenting cells and reducing CD8^+^ T cells, all of which impair the antitumoral immune response (235).

The non-selective (anti-COX 1 and 2) NSAIDs meloxicam and piroxicam have been tested *in vitro* against CMC cells, which resulted in apoptosis induction, migration inhibition, and cell cycle arrest (236, 237). Additionally, piroxicam was utilized in a xenograft model of CMC, showing a tumor size reduction (238). Further, a small group of dogs with IMC were treated with piroxicam. The mean survival time was significantly longer in dogs treated with piroxicam compared to those treated with doxorubicin-based chemotherapy (239).

Due to the availability of COX-2 selective drugs in veterinary medicine (firocoxib, deracoxib, cimicoxib, robenacoxib, and mavacoxib), their effectiveness in CMC has been studied in cell lines, mice xenografts, and, in lesser extent, in clinical trials. Celecoxib and mavacoxib have been used in CMC cell lines, showing their cytotoxic activity, proliferation inhibition, apoptosis induction, and migration reduction, even in those cell lines with low COX-2 expression, suggesting their extraordinary therapeutic applicability in COX-2-positive or COX-2-negative CMC (217, 240, 241). Deracoxib has been proven alone or in combination with piroxicam, displaying a significant decrease in cell viability, achieving an arrest in cell cycle, and induction of apoptosis in CMC cell lines (237). Additionally, when combined with doxorubicin, a strong synergistic activity was seen, allowing to reduce the dose of doxorubicin *in vitro* (242). In CMT xenografted mice, deracoxib was given in comparison to piroxicam, although its effect was lower than piroxicam. Nevertheless, the authors did not evaluate the dose of deracoxib, thus concluding that mice could have been underdosed (238).

In a clinical trial, piroxicam showed an increased in OS compared to doxorubicin in 12 IMC-bearing dogs with COX-2-positive tumor cells (239). Likewise, in a case–control prospective study, firocoxib showed higher disease-free survival and overall survival compared to mitoxantrone in dogs with highly malignant CMT-expressing COX-2 (18).

As mentioned above, in the available clinical trials on the use of NSAIDs/coxibs in CMC, patients had COX-2-positive tumors. However, *in vitro* evidence has shown that anti-COX-2 therapy may be therapeutically useful regardless of the COX-2 expression status (217).

As a conclusion, the anti-COX-2 drugs approved for their use in veterinary medicine are being used in veterinary clinics as anti-inflammatory drugs, but also as an adjuvant in neoplastic patients, no matter the expression of the enzyme in the patients' tumors and, in many cases, the lack of proper clinical trials.

### Epithelial–Mesenchymal Transition Inhibition: Cancer Stem Cells

Epithelial–mesenchymal transition (EMT) is a complex process in which epithelial cells lose their characteristics and acquire mesenchymal properties. It is essential in different embryonic stages and organ development, wound healing, and neoplastic infiltration and metastasis, cell motility, and invasiveness (243). By this process, epithelial cells undergo detachment and acquire the capacities of motility and invasiveness through the extracellular matrix to finally enter blood and lymphatic vessels and colonize different organs.

EMT is driven by the dysregulation of the adhesion molecules (mainly cadherins). Cadherins are calcium-dependent adhesion molecules responsible for cell-to-cell attachment and for maintenance of the normal structure and polarization of tissues. The most relevant are P-cadherin (placental cadherin), N-cadherin (neural cadherin), and E-cadherin (epithelial cadherin) (244). In normal human mammary tissue, E-cadherin is expressed in luminal epithelial cells, while P-cadherin is found in myoepithelial cells (245). In HBC, E-cadherin is known to be an inhibitor of metastasis, and its downregulation or inactivation leads to aggressive forms of breast cancer, EMT, lymphovascular invasion and metastasis (246, 247), as well as higher histological grade (248). Likewise, in CMC, reduction of E-cadherin expression has been related to large size and ulceration of mammary tumors (249), infiltrative growth, high histological grade, and lymph node metastasis (250–253), as well as shorter overall and disease-free survivals (254). EMT involves a range of transcription factors, including zinc finger E-box-binding homeobox 1 and 2 (ZEB1 and ZEB2), Snail, signal transducer and activator of transcription 3 (STAT3), and Twist, as well as transcriptional targets such as transforming growth factor-β (TGF-β), all of which suppress the expression of epithelial-associated molecules (E-cadherin and membranous β-catenin) and promote molecules associated with mesenchymal cell phenotypes (N-cadherin, cytoplasmic β-catenin, fibronectin, and vimentin) (255, 256). In CMC, Snail and ZEB2 have been proven to be related to E-cadherin downregulation in invasive micropapillary carcinomas (257, 258).

Not only EMT is required for successful metastatic colonization, but also cells that are capable of initiating tumorigenesis may undergo self-renewal and differentiate into various subsets of cells found in the primary tumor; these cells are called cancer stem cells (CSCs) (259). Molecular pathways that lead to EMT are markedly overlapping with those of CSC generation, allowing neoplastic epithelial cells to pass through the EMT and generate a population of CSCs (260). Additionally, CSCs are associated with therapeutic resistance (261).

Several reviews are centered on EMT as a source of metastasis, CSC generation, and therapy resistance in HBC (261–263). Likewise, studies on CMC have revealed a positive correlation between cells undergoing EMT and higher tumor grade and metastasis (264) and have identified ZEB1 and ZEB2 as potential therapeutic targets in CMT cells *in vitro*, intended to restore E-cadherin and inhibit EMT, although to date, there are no drugs targeting these molecules (265).

A number of attempts have been made for the therapeutic targeting of EMT and CSCs in HBC and CMC, which are presented below.

As aforementioned, melatonin is capable of disrupting estrogen-dependent cell signaling. In addition, *in vitro* studies on HBC and CMC cell lines have shown that melatonin is also able to reduce EMT through the degradation of β-catenin, an E-cadherin repressor (266), reducing cell migration, invasion, and CSC generation (267, 268). Significantly, the effect of melatonin is higher on ER+ CMC cells overexpressing melatonin receptors (269). In a more recent *in vitro* study, CMC cell lines treated with melatonin plus IL-25 significantly reduced cell viability, increased caspase-3-mediated apoptosis, and reduced pro-angiogenic VEGF-A (268).

Metformin, a commonly used drug in human medicine as oral treatment for type II diabetes, is being experimentally studied due to its anti-carcinogenic properties associated with the inhibition of the EMT process. Metformin has been shown to inhibit EMT in HBC cells by repressing the drivers of TGF-β (270). *In vitro* studies on CMC cell lines proved that metformin is able to induce cell cycle arrest and reduce cell migration and N-cadherin expression while increasing E-cadherin expression. The foreseen effect was ever more prominent when combined with silencing of TGF-β in the CMC cell line (271). In CMC xenografted mice treated with metformin, there was a significant decline in CSCs and reduction of lung metastasis and tumor growth (272–274). When combined with LY294002 (an inhibitor of the PI3K/AKT/mTOR pathway with anti-angiogenic properties), metformin showed a marked reduction in viability and tumor cell growth *in vitro*, while in CMC xenografted mice, both drugs decreased the tumor size and showed an important anti-angiogenic effect (reduction of VEGF-A expression and MVD) (275).

Cancer stem cells are also characterized by the presence of multidrug-resistant (MDR) adenosine triphosphate-binding cassette (ABC) transporter efflux pumps, influencing chemotherapy resistance in cancer due to their capacity to export a wide variety of cell substances (276). It has been seen that the Wnt/β-catenin pathway can upregulate the multidrug resistance protein 1 (MDR-1). Simvastatin (a lipid-lowering drug) has been studied in mammary oncology due to retrospective evidence of an improved OS and recurrence-free survival in HBC-bearing patients (277), and further *in vitro* analyses revealed an induction of apoptosis on HBC cell lines (278). *In vitro* studies using simvastatin on CMC cell lines showed that the expressions of MDR-1 and β-catenin were reduced, contributing to a chemosensitizing effect on CMC cells (279). Furthermore, when combined with doxorubicin, simvastatin exhibited a synergic cytotoxic effect on CMC cells (280). However, despite good *in vitro* results, discouraging large meta-analyses have found no significant benefit from the use of simvastatin in patients suffering from HBC (281–283).

Artemisinin, a derivate from the plant *Artemisia annua*, employed in Chinese traditional medicine, was discovered by Tu Youyou in 1972 for the treatment of malaria. By this discovery, Tu Youyou was co-recipient of the 2015 Nobel Prize in Medicine. Studies on HBC cells showed the *in vitro* suppression of N-cadherin, involved in EMT (284). Additionally, synthetic derivates of artemisinin (e.g., artesunate) are capable of inducing caspase-dependent apoptosis in HBC cells (285). The *in vitro* treatment of CMC cells with an artemisinin derivative (dihydroartemisinin, DHA) showed an inhibition of cell migration and invasiveness by downregulating the expression of EMT-related genes (Slug, ZEB1, ZEB2, and Twist) (286). In human medicine, a few phase I clinical trials have been conducted without significant adverse effects (287, 288), although a patient with HBC on artemisinin treatment showed toxic encephalopathy (289). Knowing the neurotoxicity of artemisinin in experimental animal models (290), a phase I clinical trial should be performed in domestic animals before giving any general recommendation on its use in veterinary patients.

## Immunotherapy as a Progressing Target

In human oncology, after decades of depleting the immune system of cancer patients with chemotherapy, the tendency is now to protect and increase the action of the immune system against cancer cells (291).

Cancer immunoediting is the process by which the immune system tries to destroy neoplastic cells and is composed of three steps: elimination, equilibrium, and escape (292). In the first step, the host's immune system responds to the newly formed tumor and removes it prior to any clinical evidence. If some resistant clones of the tumor are present, they survive and remain inactive in the second stage, the equilibrium. In the escape or evasion stage, tumor cells improve their ability to evade the immune system, eventually leading to clinical manifestation (293). Some of the evasion mechanisms are as follows: decreased or absent expression of major histocompatibility complex (MHC) molecules, activation of immunoregulatory pathways (immune checkpoints) such as the inhibitory molecule CTLA-4 (cytotoxic T lymphocyte antigen 4), upregulation of PD-L1 (programmed death ligand 1) that binds to the PD-1 receptor on T lymphocytes and represses their function, secretion of immunosuppressive factors such as TGF-β, interleukin-10, and VEGF, as well as induction of regulatory T cells (294). Although many of these mechanisms are already known, the true interplay between the pathways, and the interaction between tumor cells and the immune system and microenvironment, is still largely unknown.

Some mAbs that target specific molecules that intervene in a signaling pathway (e.g., anti-HER-2) have been mentioned in this review. In this section, we will discuss an intriguing class of antibodies designed to modulate the immune response and the use of vaccines, including cellular immunotherapy and DNA vaccines. Cellular immunotherapy, in particular hybrid cell vaccines, are based on cells generated by fusing antigen-presenting cells (i.e., dendritic cells) with the tumor cells of the recipient, intending to present to the immune system the whole tumor-associated antigens and activate an immune response (295). DNA vaccines are based on the introduction of one or more genes (e.g., tumor antigen, cytokines, etc.) into plasmids that are delivered into the patient with the subsequent expression of the introduced gene (296). Finally, the role of oncolytic viruses, which show selective cytotoxicity toward cancer cells and may favor the restoration of the anticancer immune function, will be reviewed (297).

The most successful human immunotherapies to date include mAbs against lymphoma antigens (i.e., CD20—rituximab) as well as mAbs against immune checkpoint molecules such as PD-1 (i.e., pembrolizumab, atezolizumab, and nivolumab) and CTLA-4 (i.e., tremelimumab and ipilimumab), which are able to release the cytotoxic activity of T lymphocytes and activate other immune responses such as antigen presentation and cytokine production (298). Besides targeting immune checkpoint molecules, other immunotherapies are being developed, such as tumor-specific cytotoxic immune cells and cytokines. However, although some cancers, such as melanoma and lymphoma, respond well to immunotherapy, other solid tumors still have weak responses (299).

Cancer cell lines and mouse models, including transgenic mice and patient-derived xenografts, have been extremely useful in the study of human cancer, yielding valuable insights into cancer biology, genetics, and biochemistry (300). However, they have limitations and lack essential features inherent only to spontaneous cancers, like an intact complex immune system (301, 302). Because normal immunocompetent mice reject human tumor grafts, there are no preclinical experimental models to investigate immunotherapy for cancer patient tumors. Canine patients with CMC have been used as an intermediate model in several clinical trials with novel immunotherapy (303).

Several clinical trials have revealed relevant information regarding canine cancer immunotherapy in osteosarcoma, lymphoma, melanoma, meningioma, bladder cancer, soft tissue sarcoma, and hemangiosarcoma using multiple immunotherapeutic approaches (DNA vaccines, cellular immunotherapy, mAbs, bacteria, etc.) (303–310), which are out of the scope of this review.

### Antibody Immunotherapy

Even though HBC is not considered a highly immunogenic cancer, immunotherapeutic strategies are being successfully tested especially against TNBC (311). Proof of this is that the FDA approved an immunotherapy for advanced PD-L1+ TNBC, atezolizumab (anti-PD-1 mAB), in combination with paclitaxel (312). Another anti-PD-L1 mAb, pembrolizumab, has also been shown to improve the progression-free survival in PD-1+ TNBC when combined with paclitaxel, gemcitabine, carboplatin, or eribulin (313, 314). Although PD-1 has been detected in CMTs (315, 316), no attempt has been made to use anti-PD-1 or anti-PD-L1 mAbs against CMC, perhaps due to cost restrictions. The only published clinical trial with mAbs against CMC (including IMC) used the mouse monoclonal antibody BR96, which recognizes a specific antigen (Lewis^y^-related carbohydrate, Le^y^) expressed in several solid tumors and gastrointestinal epithelium, conjugated with a truncated, non-binding derivate of *Pseudomonas* exotoxin A. Stable disease or partial response was achieved in IMC cases, as well as neutralizing antibodies (317). In addition to this, in 2014, a canine anti-EGFR-1 mAb was developed, but it never reached the clinical or preclinical level (151).

### Cellular Immunotherapy

Cellular immunotherapy is an emerging field of research in which immune cells are extracted, modified, and reinfused into the patient (318). In HBC, two main types of cellular immunotherapy have been studied: adoptive cell therapy (ACT, based on T lymphocytes) and dendritic cell therapy (319). ACT is based on the isolation of T lymphocytes in the resected tumor, *in vitro* expansion or modification, and subsequent reinfusion (318). Few information on HBC are available to date. Direct T lymphocyte reinfusion in combination with pembrolizumab led to a complete durable regression in a patient with chemotherapy-refractory HBC (320). Chimeric antigen receptor (CAR) T cell therapy is a type of modification where the receptor is engineered to target a specific antigen and combine antigen-binding and T cell-activating functions, hence recognizing antigens in the absence of the presentation by the MHC (321). To date, only preclinical studies have been published using HER-2, mucin 1 cell surface associated (MUC-1), mesothelin (MSLN), epithelial cell adhesion molecule (EPCAM), and carcinoembryonic antigen (CEA) as targets (322). On the other hand, in dendritic cell therapy, the cells are isolated and combined with tumor antigens before reinfusion into the patient (319). Despite clinical settings being currently limited to phase I/II human trials, preclinical studies on HER-2-loaded and cyclin D1-loaded dendritic cell vaccines have been shown to significantly inhibit the HBC xenografted tumor growth in mice (323). Recently, autologous hybrid-cell vaccines were produced for a clinical trial in CMC as an intermediate model for HBC. The therapy was combined with immunostimulatory oligonucleotides and gemcitabine and achieved 3.3 times longer median survival times than the control group, except for the case of IMC, which only resulted in a median of 42 days (324).

### DNA Vaccines

DNA, or gene-based, vaccines are intended to deliver functional genes to the target cells for the expression of functional proteins (325). Since naked DNA is readily accessible for endonucleases, the DNA needs an effective and safe delivery method, which can be biological (e.g., viruses and bacteria) or non-biological (e.g., physical methods such as electroporation or chemical methods such as nanoparticles) (326). In HBC, different delivery methods have been tested to transport tumoral antigens, such as HER-2, *p*53, MUC1, Twist, and mammaglobin-1, as well as immunostimulatory molecules such as IL-6 and IL-12 (326, 327).

Since the intravenous administration of IL-12 has been associated with grave toxicity in humans (328), a clinical trial using nine dogs, one of them with a mammary tumor, used intratumoral IL-12 (plasmid DNA by electroporation) as an immunostimulatory cytokine. Despite transient increases in serum and tumor IL-12 and IFN-⋎, no clinically relevant outcome benefits were seen (329). Similarly to this, recombinant viral vaccines, based on replication-defective recombinant adenoviruses, have been proven to be safe and to induce strong antibody and cellular antigen-specific immune responses in non-human primates (330). A study evaluated the ability of DNA electroporation and a recombinant adenovirus serotype 6, both expressing telomerase reverse transcriptase (overexpressed in tumor cells, while low to absent in normal cells) and HER-2, to induce immune responses in healthy dogs against these proteins. A detectable and long-standing cellular and humoral immune response was detected in the absence of side effects or autoimmunity (331).

An anticancer DNA vaccine based on p62 (a protein involved in selective macroautophagy that is dispensable for most tissues, but essential for the development and survival of tumors) was utilized in CMC xenografted mice and dogs bearing mammary carcinomas. The intramuscular administration of the *p*62 DNA vaccine achieved a partial response or stable disease in the absence of noteworthy secondary effects. Antitumoral activity was related to lymphocyte infiltration, particularly T lymphocytes, and tumor encapsulation *via* fibrosis (332, 333).

Another clinical trial with dogs presenting CMC utilized nanoparticles carrying DNA plasmids coding canine interferon-β and herpes simplex virus (HSV) thymidine kinase (a suicide gene), which were injected into the tumor bed during mastectomy; afterwards, subcutaneous injections of the nanoparticles associated with a human granulocyte–macrophage colony-stimulating factor and interleukin-2, mixed with allogeneic mammary carcinoma extracts, were periodically administered. The therapy was well-tolerated; only one out of 26 patients had recurrence and none displayed distant metastasis, and overall survival was also improved (334).

### Oncolytic Viruses

Oncolytic viruses show selective cytotoxicity toward cancer cells and may favor the restoration of immune anticancer function (297). Clinical trials with oncolytic viruses are currently ongoing in humans, and two viruses have been approved for commercial use. The first, H101 or Oncorine®, is an adenoviral construct with an E1B deletion (in order to avoid replication in normal cells), approved in China in 2005 for the treatment of head-and-neck squamous cell carcinoma (335). The second, T-VEC or Imlygic^TM^, is an engineered herpes simplex virus type I (HSV-1) that expresses the human granulocyte–monocyte colony-stimulating factor as an immune stimulant, approved in 2015 by the United States FDA for the local treatment of unresectable cutaneous, subcutaneous, and nodal lesions in patients with recurrent melanoma after initial surgery (336). In veterinary medicine, a number of viruses with natural oncolytic capacity, as well as engineered viruses, are being studied for several neoplasms (337, 337–349). Among them are morbillivirus, poxvirus, and reovirus.

#### Oncolytic Morbillivirus

In human medicine, the measles virus has demonstrated an oncolytic potential since anecdotical reports describing the regression of hematopoietic neoplasms after natural infection with measles virus (350). In HBC, the measles virus has shown a strong cytolytic effect in cancer cell lines *in vitro* (351–354). Additionally, attenuated measles virus has been proven to overcome chemoresistance in HBC cells. Several studies in mice, however, showed that viral replication also existed in the organs and cells of infected mice and not only in the targeted tumor cells (355–357). Therefore, some modifications have been done to the viral strains. For instance, a recombinant measles virus strain was created by eliminating its ability to bind to the signaling lymphocyte activation molecule (SLAM), a major receptor used by the wild-type virus to infect immune cells in naturally occurring infections. Cancer cells do not express SLAM molecules, but the measles virus is able to use the Nectin-4 receptor to bind and infect the cell (351). Interestingly, Nectin-4 expression has been found in HBC cells (358) and in 45% of CMT tissue samples and in CMC cell lines. Recombinant measles virus strain exerts cytotoxic effects in Nectin-4-expressing CMC cell lines. In Nectin-4-expressing CMC xenografted mice, this oncolytic therapy showed significant suppression of tumor growth without any noticeable adverse effect (340).

Canine distemper virus (CDV) has been shown to induce apoptosis in the cerebellum and lymphoid tissue of naturally infected dogs through the extrinsic pathway, activating caspase-8 and caspase-3 (359, 360). Therefore, this morbillivirus is considered to be a candidate for potential treatment in canine malignancies. An attenuated strain of CDV showed a substantial oncolytic effect in CMC cells both *in vitro* and in xenografted mice, without significant adverse events, by inducing apoptosis through the same pathways as natural infection (extrinsic pathway) with participation of nuclear factor kappa light-chain enhancer of activated B cells (NF-κB) (346, 361).

#### Oncolytic Poxvirus

Several studies on the use of multiple strains of vaccinia virus (362–366) have demonstrated the *in vitro* and preclinical effects against HBC, which have led to a randomized phase III clinical trial in patients with metastatic HBC in which the use of a poxviral vaccine in combination with docetaxel resulted in an increase in progression-free survival (367).

Two oncolytic strains of vaccinia virus (strain GLV-1h68 and strain GLV-5b451 expressing GLAF-2, an antibody against VEGF) have been tested against CMC cells *in vitro* or in xenografted mice, resulting in efficient infection and lysis of cells *in vitro* while achieving significant tumor growth inhibition *in vivo* with strong inflammatory and oncolytic-associated effects (368, 369) and a reduction of MVD in the tumors treated with strain GLV-5b451 (337).

An attenuated form of *Myxoma virus* lacking the *serp2* gene (an anti-apoptotic virulence factor) was used to evaluate its oncolytic activity in canine mammary cancer cells and showed severe cytopathic effects and adequate viral replication (370).

#### Oncolytic Reovirus

The oncolytic virus pelareorep (REOLYSIN®) is a non-modified serotype 3 reovirus strain that has shown antitumor activity in clinical and preclinical models, especially in pancreatic cancer, and currently is being tested in clinical trials to assess its efficacy as an oncolytic agent against several cancers. In HBC cells, REOLYSIN® infection in the presence of DNA-damaging agents enhances infection and triple-negative breast cancer cell killing by the reovirus (371).

REOLYSIN® was tested in CMC cells *in vitro* and in xenografted mice, demonstrating significant cell death *via* caspase-3-mediated apoptosis (338). When combining this oncolytic therapy with low doses of paclitaxel, carboplatin, gemcitabine, or toceranib, its activity was enhanced with all the therapeutic agents, except toceranib (341). A series of cases of dogs with various malignancies, which included two cases of CMC (one IMC and one non-IMC), were treated with intratumoral or intravenous REOLYSIN®. Less than 50% of the dogs presented grade I or II adverse effects, which included vomiting, diarrhea, and inflammation of the injected tumor. Dogs did not shed virus and had elevated neutralizing antibodies. No specific information about the antitumoral response in CMC patients was provided (341, 345).

## Other Adjuvant Therapies

### Nanotechnology

Nanotechnology has been evolving rapidly in recent years, providing new therapeutic tools for several diseases. Nanoparticles are defined as particles below 100 nm of dimension, although their surface is generally large enough to bind and carry therapeutic compounds (372). Nanoparticles have been proposed as drug carriers in cancer treatment since they can increase drug accumulation in target tissues, optimizing the therapeutic effect (373).

Several nanoparticle-based delivery platforms have been approved by the US FDA, and two nano-based drugs are already in the market for HBC—Doxil® (doxorubicin-loaded nanoparticles) and Abraxane® (albumin-bound paclitaxel-loaded nanoparticles)—whose coating evades the immune system, allowing a precise targeting delivery (374). Additionally, several clinical trials with HBC patients are currently under study using these delivery platforms to carry doxorubicin, paclitaxel, cisplatin, irinotecan, annamycin (synthetic derivate of doxorubicin), and docetaxel (375–379).

In dogs, doxorubicin is a commonly used chemotherapeutic for CMC. However, its toxicity is dose-limiting, reducing treatment efficacy (58). Aldoxorubicin (a prodoxorubicin bound to albumin, which is cleaved from the drug in the acidic tumor microenvironment) (380) was constructed into nanofiber peptides and administered parenterally to xenografted mice bearing HBC, reducing primary tumor burden and lung metastasis as well as improving survival (381). This was also tested in canine mammary tumor cells *in vitro*, showing an excellent anti-proliferative effect at lower doses than free aldoxorubicin or doxorubicin (382).

Gold nanoparticles, associated with other minerals, have been used in human oncology as an aid in diagnostic methods (due to the molecular weight of gold, it captures many x-rays) and as support in thermo- and phototherapy (383). However, direct *in vitro* toxicity has been seen against human colorectal, hepatocellular, and mammary carcinoma cell lines (384). Two metal compounds, Co(III) and Zn(III), that have previously shown remarkable anti-proliferative activity against human colorectal carcinoma cells, hepatocellular carcinoma cells, and breast carcinoma cells were loaded onto 14-nm gold nanoparticles. These metal compounds demonstrated to efficiently lyse CMC cells *in vitro*; moreover, when loaded onto nanoparticles, the effect was even stronger at lower doses (385).

### Herbal Medicine

Herbal medicine has long been administered to treat malignancies in Asian countries (386). While 86.4% of HBC patients with Asian background tend to use herbal medicine, the figures in the Western world, although significantly lower, are continually increasing (387). Over the years, numerous herbal compounds with anticancer activities, such as proliferation inhibition, apoptosis induction, anti-angiogenic, and anti-metastatic, have been identified. The most common natural products are curcumin, berberine, artemisinins, ginsenoides, ursolic acid, silibinin, emodin, triptolide, cucurbitacins, tanshinones, ordonin, shikonin, gambogic acid (GA), artesunate, wogonin, β-elemene, and cepharanthine (388).

Few phytochemicals have been studied in veterinary oncology. The following are those related to CMC.

Curcumin is a phytochemical isolated from the rhizome of turmeric (*Curcuma longa*), used in traditional medicine in India and China for a long time (389). *In vitro* studies have demonstrated its anti-proliferative, pro-apoptotic, anti-angiogenic, and chemosensitizing activities against several tumor types, including breast cancer cells (390–396). In veterinary medicine, curcumin and carnosic acid, derived from rosemary leaf extract, were used alone or in combination on mastocytoma, osteosarcoma, and CMC cell lines and resulted in caspase 3 and 7 activation and apoptosis, with a potent synergistic effect when used in combination (397). A major limitation of curcumin is its low absorption (< 1% of orally administered curcumin will be absorbed) (398). Therefore, a liposome-encapsulated curcumin formulation that enables intravenous delivery was developed and tested on canine cancer cells such as CMC, melanoma, and osteosarcoma cell lines, as well as endothelial cells. Cell proliferation was effectively inhibited with the treatment; likewise, the viability, migration, and tube formation of endothelial cells were suppressed. In the same study, a pilot clinical trial was conducted with cancer-bearing dogs (CMC, pulmonary carcinoma, thyroid carcinoma, chest wall sarcoma, osteosarcoma, and malignant melanoma), achieving stable disease in ~60% of dogs (399). Finally, a combination of curcumin and paclitaxel was loaded into silica nanoparticles and delivered into CMC cell lines, manifesting a clear and persistent cytotoxic effect (400, 401).

The edible wild ginger *Zingiber zerumbet* contains several phytochemicals with healing properties; zerumbone is one of the most important due to its antitumor, anti-inflammatory, antioxidant, antimicrobial, antinociceptive, hepatoprotective, and immunomodulatory activities (402). However, its poor absorption and bioavailability are the main issues for its therapeutic application (403). Therefore, nanostructured lipid carriers loaded with zerumbone have been used as an apoptogenic agent in several neoplastic human and canine cell lines, including CMC. The effect was attributable to increases in caspase-8, caspase-9, caspase-3, and caspase-7 (404).

The last one of the herbal compounds that have been used for canine mammary tumors is berberine, an isoquinolone alkaloid of the plant *Berberis vulgaris* L. that inhibited the proliferation of CMC cells *in vitro* (405).

### Old Drugs as New Therapies

Ivermectin is a well-known anti-parasitic agent used to treat a variety of canine parasitic infestations. The mechanism of action of ivermectin in parasites is due to blockade of the parasite chloride channel (406). Currently, ivermectin has been linked to a potential anticancer effect in different tumor types, including breast cancer (407). *In vitro* studies using ivermectin in CMC cell lines, and further in xenografted mice, effectively inhibited cell growth in a dose- and time-dependent manner. The effects were associated with cell cycle arrest *via* the downregulation of CDK4 and cyclin D1 expressions and reduced WNT/β-catenin signaling (408).

Selenium possesses different anti-neoplastic mechanisms: promotion of cell apoptosis, anti-angiogenesis, and immune system regulation. Also, its antioxidant effect may reduce the toxicity of conventional chemotherapeutics if used in combination (409). Different selenium compounds (sodium selenite, methylseleninic acid, and methylselenocysteine) showed *in vitro* anti-proliferative effects on CMC that were even greater when combined with cyclophosphamide. An increase of apoptosis, downregulation of pro-angiogenic VEGFA, angiopoietin-2, and hypoxia-inducible factor-1 alpha, and upregulation of the anti-angiogenic and anti-proliferative phosphatase and tensin homolog (PTEN) were the major features (409). In further CMC xenografted mouse models, the different selenium compounds significantly inhibited tumor growth, generated large necrotic areas, and reduced the MVD compared to the untreated control. This *in vivo* study also found a reduction in pro-angiogenic factors (VEGFA, PDGF, and angiopoietin-2) (410).

Salinomycin is an ionophore antibiotic isolated from *Streptomyces albus*, which is widely used in farm animals as an anticoccidial drug (411). Several studies with HBC cell lines demonstrated that salinomycin inhibits *in vitro* growth by inducing apoptosis and selectively targeting CSC (412–419). Likewise, salinomycin was found to have a profound effect in CMC cell lines by selectively depleting canine mammary CSC and inhibiting the Wnt/β-catenin signaling pathway (preventing cell invasion and migration) (420, 421).

## Discussion

A total of 71 studies focused on adjuvant therapies in CMTs, not including those related to surgery or conventional chemotherapy, were analyzed in this review. The majority of those studies were performed *in vitro* (49 studies); 15 used xenografted mice to study CMC (in total, 15 papers of mouse models of CMTs). Only six of those investigations done with CMC cells *in vitro* or in mouse models, have reached the clinical setting (138, 143, 239, 332, 333, 341, 345, 399).

Setting clinical studies of conventional chemotherapy aside, to date, 18 clinical trials have been conducted in dogs with CMC, and a third of them are new immunotherapies, which demonstrate the usefulness of spontaneous CMC as a natural model for the study of HBC in a natural model with a complete immune system. They are also a reflection of the current state of cancer research and the important trend to stimulate the immune system against cancer cells (291). In spite of the relevance of such investigations in dogs, the number of patients recruited in them is very low to obtain conclusive and extrapolative results: five of the 18 clinical studies were done with less than eight dogs with mammary cancer; four of them with less than three dogs. In addition, two more studies were executed in non-tumor-bearing dogs. As a main conclusion, larger prospective randomized studies are needed to provide a strong level of evidence that allows a widespread use of some of these new approaches. Considering that CMTs are the most common malignancy in dogs and the low rate of success of routine adjuvant therapies (i.e., conventional chemotherapy), these clinical trials seem to not be enough. Significantly greater effort must be made to generate knowledge and develop canine-specific targeted therapies. There is a great need for well-planned large prospective randomized clinical trials in dogs with CMC to obtain valid results for both species, humans and dogs, on the use of new therapies.

Following the One Health concept, human and veterinary oncology will have to join forces to take advantage of both the economic and technological resources that are invested in HBC research, together with the innumerable advantages of dogs with CMC as a spontaneous animal model.

## Author Contributions

LP and GV devised, structured, and wrote the manuscript. GV reviewed the literature. ÁA-D and GV performed the immunohistochemical slides and the photographs. All the authors reviewed and corrected the manuscript.

## Conflict of Interest

The authors declare that the research was conducted in the absence of any commercial or financial relationships that could be construed as a potential conflict of interest.
